# Aquaporins are main contributors to root hydraulic conductivity in pearl millet [*Pennisetum glaucum* (L) R. Br.]

**DOI:** 10.1371/journal.pone.0233481

**Published:** 2020-10-01

**Authors:** Alexandre Grondin, Pablo Affortit, Christine Tranchant-Dubreuil, Carla de la Fuente-Cantó, Cédric Mariac, Pascal Gantet, Vincent Vadez, Yves Vigouroux, Laurent Laplaze

**Affiliations:** 1 UMR DIADE, IRD, Université de Montpellier, Montpellier, France; 2 Laboratoire Mixte International Adaptation des Plantes et Microorganismes Associés Aux Stress Environnementaux, Dakar, Senegal; 3 Laboratoire Commun de Microbiologie, Dakar, Senegal; 4 Centre d’Étude Régional pour l’Amélioration de l’Adaptation à la Sécheresse, Thiès, Senegal; 5 International Crops Research Institute for Semi-Arid Tropics (ICRISAT), Hyderabad, India; University of Tasmania, AUSTRALIA

## Abstract

Pearl millet is a key cereal for food security in arid and semi-arid regions but its yield is increasingly threatened by water stress. Physiological mechanisms relating to conservation of soil water or increased water use efficiency can alleviate that stress. Aquaporins (AQP) are water channels that mediate root water transport, thereby influencing plant hydraulics, transpiration and soil water conservation. However, AQP remain largely uncharacterized in pearl millet. Here, we studied AQP function in root water transport in two pearl millet lines contrasting for water use efficiency (WUE). We observed that these lines also contrasted for root hydraulic conductivity (Lpr) and AQP contribution to Lpr. The line with lower WUE showed significantly higher AQP contribution to Lpr. To investigate AQP isoforms contributing to Lpr, we developed genomic approaches to first identify the entire AQP family in pearl millet and secondly, characterize the plasma membrane intrinsic proteins (PIP) gene expression profile. We identified and annotated 33 AQP genes in pearl millet, among which ten encoded PIP isoforms. *PgPIP1-3* and *PgPIP1-4* were significantly more expressed in the line showing lower WUE, higher Lpr and higher AQP contribution to Lpr. Overall, our study suggests that the PIP1 AQP family are the main regulators of Lpr in pearl millet and may possibly be associated with mechanisms associated to whole plant water use. This study paves the way for further investigations on AQP functions in pearl millet hydraulics and adaptation to environmental stresses.

## Introduction

Plant hydraulics depends on soil water capture by roots, transport to the leaves and diffusion as vapor from the stomatal cavity to the atmosphere. In this plant hydraulic continuum, radial water transport from the soil solution to the xylem vessels uses two paths: the apoplastic path where water flows along the cell wall structures, and the cell to cell path where water can flow across cell membranes (transcellular) or along cytoplasmic continuities formed by plasmodesmata (symplastic) [1]. Extracellular hydrophobic barriers of lignin and suberin located in the endodermis are thought to restrict diffusion of water along the apoplastic path [2]. In the presence of such barriers, water channels present in cell membranes called aquaporins enable cell to cell transport [3].

Aquaporins (AQP) are present across life forms, with the exception of thermophilic Archaea and intracellular bacteria [4]. They belong to the Major Intrinsic Proteins (MIP) superfamily and are characterized by six transmembrane domains and two highly conserved Asn-Pro-Ala (NPA) motifs [5]. Another typical AQP signature is selectivity filters structuring the pore, which are composed of aromatic/arginine (ar/R) motifs and Froger’s residues [6, 7]. In higher plants, AQP isoforms fall into five families; Plasma membrane Intrinsic Proteins (PIP), Tonoplast Intrinsic Proteins (TIP), Nodulin26-like Intrinsic Proteins (NIP), Small Intrinsic Proteins (SIP) and uncharacterized (X) Intrinsic Proteins (XIP) [8]. Although plant AQP are localized throughout the cell secretory system, PIP, NIP and XIP are preferentially located in plasma membranes, while TIP accumulate in the tonoplast and SIP in the endoplasmic reticulum [9–11]. Functional studies, combined with modelling approaches demonstrated that in addition to being permeable to water, AQP also transport other substrates [12, 13]. Some PIP isoforms are permeable to hydrogen peroxide (H_2_O_2_), nitrate (NO_3_^−^) and carbon dioxide (CO_2_), some TIP isoforms to ammonia (NH_3_) and urea, and some NIP to small organic solutes or mineral nutrients [14–19]. AQP possess wide range of physiological functions and have now been identified in a number of crop species such as rice, maize, tomato, cotton, sorghum, foxtail millet, watermelon and cannabis [20–25].

In plants, AQP are involved in water transport in both roots and shoots. In Arabidopsis, isoforms AtPIP2-2 and AtPIP1-2 contribute around 14% and 20% of the root osmotic conductivity and shoot hydraulic conductivity, respectively [26, 27]. AQP also have important roles in plant growth, CO_2_ fixation, nutrient allocation, reproduction or biotic interactions [8]. In recent years, AQP functions in plant water relations have received more attention as a potential target for crop improvement [28–33]. For instance, AQP could contribute to balancing root water transport with transpiration in soybean under high evaporative demand [34]. This hypothesis is supported by the increased AQP expression and root hydraulic conductivity upon high transpiration demand in rice and grapevine [35, 36]. Furthermore, overexpression of *SiTIP2-2* in tomato increased transpiration rate and was associated with improved fruit yield upon moderate soil water stress [37]. Conversely, in other crops more adapted to hot and dry climates such as pearl millet, lower transpiration under high vapor pressure deficit (VPD) has been associated with AQP function and proposed to be beneficial for crop yield under such conditions [31, 38, 39]. Therefore, AQP may be involved in different physiological mechanisms that determine the rate and pattern of plant water usage [40]. In fact, specific isoforms might be involved in different scenarios, calling for a better understanding of AQP family members and their functions in crops [29].

Pearl millet [*Pennisetum glaucum* (L) R Br.] is a key cereal for food security in arid and semi-arid regions [41]. However, its yields are low and are often affected by climate unpredictability (heat waves and dry spells), which is forecast to worsen [42, 43]. The recent release of the pearl millet genome has opened new opportunities for functional genomic-based efforts to improve pearl millet yield and abiotic stress tolerance [44]. In this study, links between AQP function in roots and water use were investigated by measuring AQP contribution to Lpr in two pearl millet inbred lines contrasting for water use efficiency. The entire AQP family in pearl millet was characterized using a genomic approach and analyses of root AQP gene expression provided insights into how AQP isoform contribute to root hydraulics.

## Materials and methods

### Plant material and growth conditions

IP4952 and IP17150, two pearl millet inbred lines that are part of the Pearl Millet inbred Germplasm Association Panel (PMiGAP) were used in this study [44]. The water use efficiency (expressed as a plant biomass produced per amount of water transpired in g.kg^-1^) of these two lines was characterized in two lysimeters experiments performed under well-irrigated conditions at the International Crops Research Institute for the Semi-Arid Tropics (ICRISAT, India) using the protocol outlined in [45]. These experiments indicated that IP4952 and IP17150 displayed relatively low and high water use efficiency, respectively (2.35 versus 3.72 g.kg^-1^) [46]. These two lines were used for root hydraulic conductivity measurements and AQP expression analyses in which plants were grown in hydroponic conditions in a nethouse (greenhouse made of shade nets) at the IRD/ISRA Bel Air research station (Dakar, Senegal; 14.701615 N– 17.425359 W). Plants were germinated in Petri dishes in a dark chamber at 37°C for two days. Plants were exposed to light (37°C, 12h day/night cycle) for one day before being transplanted to a black mat covering a 30L container (45x40x24 cm containing 40 plants in total) filled with half strength Hoagland solution [47]. The system allowed roots to grow in the nutrient solution without been exposed to light. Oxygen was constantly supplied to the root zone using an air pump.

### Root hydraulic conductivity and aquaporin contribution

Root hydraulic conductivity (Lpr) was measured in April-May 2019 from 9AM to 12PM on 15 day old plants. Minimum and maximum temperature and humidity over the period of plant growth was 17/34°C and 34/100%. At the time of measurements, average temperature, relative humidity and VDP were 24.4 ± 0.3°C, 75.1 ± 1.2% and 0.8 ± 0.1, respectively. Plants replicates were grown sequentially to allow analysis at the same age, in a randomized design taking into account the time of measurement. Lpr measurements were performed using a pressure bomb (model 1000, PMS instrument company, USA) according to [48] and [49]. Briefly, plants were inserted into the pressure chamber filled with nutrient solution with our without 2mM azide (NaN_3_). The hypocotyl was carefully threated through the silicone grommet of the pressure chamber lid so the intact root system was sealed into the chamber. Roots were pressurized with compressed air at 0.4 MPa for 5 min to equilibrate, followed by xylem sap collection at 0.1, 0.2 and 0.3 MPa for 5 min using pre-weighed 2 ml Eppendorf tubes filled with cotton placed on top of the stem. The mass of xylem sap exuded at each pressure was determined by weighing and was used to calculate root conductance (L_0_; slope of xylem sap weight at each pressure). After the measurements, roots systems were scanned to determine average root diameter, length and root surface area using WinRhizo Pro version 2012b (Regent Instruments, Canada). Root conductance was divided by root surface area to calculate Lpr. AQP contribution to Lpr was estimated using relative Lpr inhibition by azide calculated as:

$$100–\left(\left(Lpr_{azide\_individual\_replicate}\;x\;100\right)/Lpr_{no\_azide\_variety\_mean}\right)$$ (1)

### Genome-wide aquaporin identification

A total of 772 AQP protein sequences from 19 plant species (*Arabidopsis thaliana*, *Beta vulgaris*, *Brachypodium distachyon*, *Cicer arietinum*, *Gossypium hirsutum*, *Glycine max*, *Hordeum vulgare*, *Linum usitatissimum*, *Musa acuminate*, *Panicum virgatum*, *Pennisetum glaucum*, *Populus tremula*, *Oryza sativa*, *Setaria italica*, *Solanum lycopersicum*, *Solanum tuberosum*, *Sorghum bicolor*, *Vitis vinifera* and *Zea mays*) were aligned against the pearl millet genome (ASM217483v2) and the non-assembled pearl millet scaffolds [44] using tblastn with an e-value of 10^−5^ as initial cut-off to identify high scoring pairs. High scoring pairs were further filtered to keep those with a bit score ≥100. Hot-spots of high scoring pairs were identified and redundant high scoring pairs were filtered to keep those with highest bit-score for further analysis (S1 Table). The locations of filtered high scoring pairs in the pearl millet genome were used to identify regions with homologies to AQP genes.

### Structural annotation

Correspondence between selected high scoring pairs and annotated genes in the pearl millet genome was investigated. Potential AQP genes were identified and their genomic sequence ± 1000pb upstream and downstream of the start/end position was retrieved as well as the predicted gene structure [44]. When predicted high scoring pairs did not correspond to previously annotated genes, the GENSCAN Web Server (http://hollywood.mit.edu/GENSCAN.html) was used to predict the exon-intron structure of the genomic region. Putative AQP genomic sequences were aligned against the Plant EST (downloaded in August 2018) and the UniProt/Swiss-Prot plant protein (February 2016) databases and were manually annotated using the Artemis software (version 17.0.1, Sanger Institute, UK; S2 Table). Annotation was confirmed by aligning reads from pearl millet transcriptomes [50] against the pearl millet genome using the Tablet software (version 1.19.09.03) [51]. Coding and protein sequences were then generated. AQP gene structure were visualized using GSDS2.0 software [52]. The genomic, coding and protein sequences of the pearl millet AQP genes are available at the following address: https://dataverse.ird.fr/dataset.xhtml?persistentId=doi:10.23708/WVCG5O.

### Sequencing

AQP genes with incomplete sequences in coding regions were resequenced (S2 Table) using genomic DNA or cDNA from Tift 23D2B1 (genotype used to draft the pearl millet whole genome sequence). DNA was prepared using DNeasy Plant mini extraction kit (Qiagen, Germany). cDNA was prepared by first extracting RNA using the RNeasy Plant mini extraction kit (Qiagen, Germany), followed by DNAse treatment (RNase-free DNase set; Qiagen, Germany) and then reverse transcription reaction (Omniscript RT kit; Qiagen, Germany), according to the manufacturer’s instructions. Corresponding DNA/cDNA fragments were amplified using the Phusion high-fidelity DNA polymerase (Thermo Scientific, USA), purified (Geneclean turbo kit, MP Biomedicals, USA) and sent for sequencing (Eurofins Genomics, Germany). These new sequences are referenced in Genbank under the accession number MT474859 for *Pennisetum glaucum* (Pg) PIP2-8, MT474860 for PgTIP3-1, MT474861 for PgTIP4-1, MT474862 for PgTIP4-2, MT474863 for PgTIP4-3, MT474866 for PgNIP1-2, MT474867 for PgNIP3-5, MT474868 for PgSIP1-2 and MT474869 for PgSIP2-1. Primers used for amplification are presented in S3 Table. Difficulties in amplifying the missing sequence of PgTIP5-1 were encountered. In that specific case, unpublished MINION reads (Mariac, Vigouroux, Berthouly-Salazar; unpublished) were used to complete its sequence (Genbank accessions MT474864 and MT474865). Missing nucleotides were assembled onto the pearl millet genomic sequence and the coding frame of the new protein was determined.

### Identification of functional motifs and transmembrane domains

The NCBI conserved domain database (CDD) was used to identify Asn-Pro-Ala (NPA) motifs and aromatic/arginine (ar/R) selectivity filters in the putative AQP protein sequences. Froger’s residues were identified according to [6]. The number and location of the transmembrane domains were studied using TMHMM (http://www.cbs.dtu.dk/services/TMHMM/), TMpred (https://embnet.vital-it.ch/software/TMPRED_form.html) and Phyre2 [53]. Protein sequences were aligned using the CLUSTALW alignment function in the Maga7 software (version 7.0.26) [54]. Alignments were colored using the Color Align Properties program (https://www.bioinformatics.org/sms2/color_align_prop.html). Conserved domains, as well as transmembrane domains, were further manually analyzed to detect sequence alterations. Phyre2 was also used to predict *Pennisetum glaucum* PIP (PgPIPs) structure based on homologies with aquaporins of known structure. This generated Protein Data Bank (PDB) files that were used for studying three-dimensional geometric structure and pore morphology of PIP using PoreWalker software (https://www.ebi.ac.uk/thornton-srv/software/PoreWalker/). The PDB files are available at the following address: https://dataverse.ird.fr/dataset.xhtml?persistentId=doi:10.23708/WVCG5O.

### Phylogenetic analysis

Phylogenetic analyses of *P*. *glaucum* AQP (PgAQP) was conducted including AQP identified in *A*. *thaliana* (AtAQP), *O*. *sativa* (OsAQP) and *P*. *tremula* (PtAQP) using the Mega7 software (version 7.0.26) [54]. PgAQP, AtAQP, OsAQP and PtAQP protein sequences were aligned using the CLUSTAW function and a phylogenetic tree was built using the maximum likelihood method with 1000 reiterations. This allowed determination of the statistical stability of each node. Based on their position in the phylogenetic tree, PgAQP isoforms were classified into PIP, SIP, TIP and NIP families and named according to their close homologs.

### Expression profiling

Quantitative polymerase chain reaction (RT-PCR) was used to measure PgPIP gene expression in 15 day old plants grown in hydroponic conditions. One seminal and one crown root were collected between 10AM and 12PM. Sampled roots were immediately frozen using liquid nitrogen and later ground using a TissueLyser II (Qiagen). RNA and cDNA (from 1μg of RNA) were prepared using extraction kits as described above. RT-PCR was performed with 1μL of diluted cDNA (1:9) in a Brillant III ultra fast SYBRgreen QPCR master mix (Agilent Technologies, USA) using a StepOnePlus Real-Time PCR System (Applied biosystems, USA). Primers used to amplify the different PgPIP genes were checked for specificity and efficiency prior to the experiment (S4 Table). The pearl millet Ubiquitin gene (*Pgl_GLEAN_10001684*) was used as a reference and PgPIP relative expression was calculated according to the delta-delta ct method.

Data to characterize PgAQP shoot expression profiles were collected from [50]. Leaves and inflorescence of ten open pollinated cultivated pearl millet varieties were used [50].

### Statistics

Statistical analyses were performed using R version 3.5.2 (R Development Core Team, 2018) using ANOVA (aov script) to detect significant differences. The Least Significant Difference (LSD) test, within the Agricolae package, was used to group differences in letter classes.

## Results

### Pearl millet aquaporin contribution to root hydraulic conductivity

**Fig 1:**
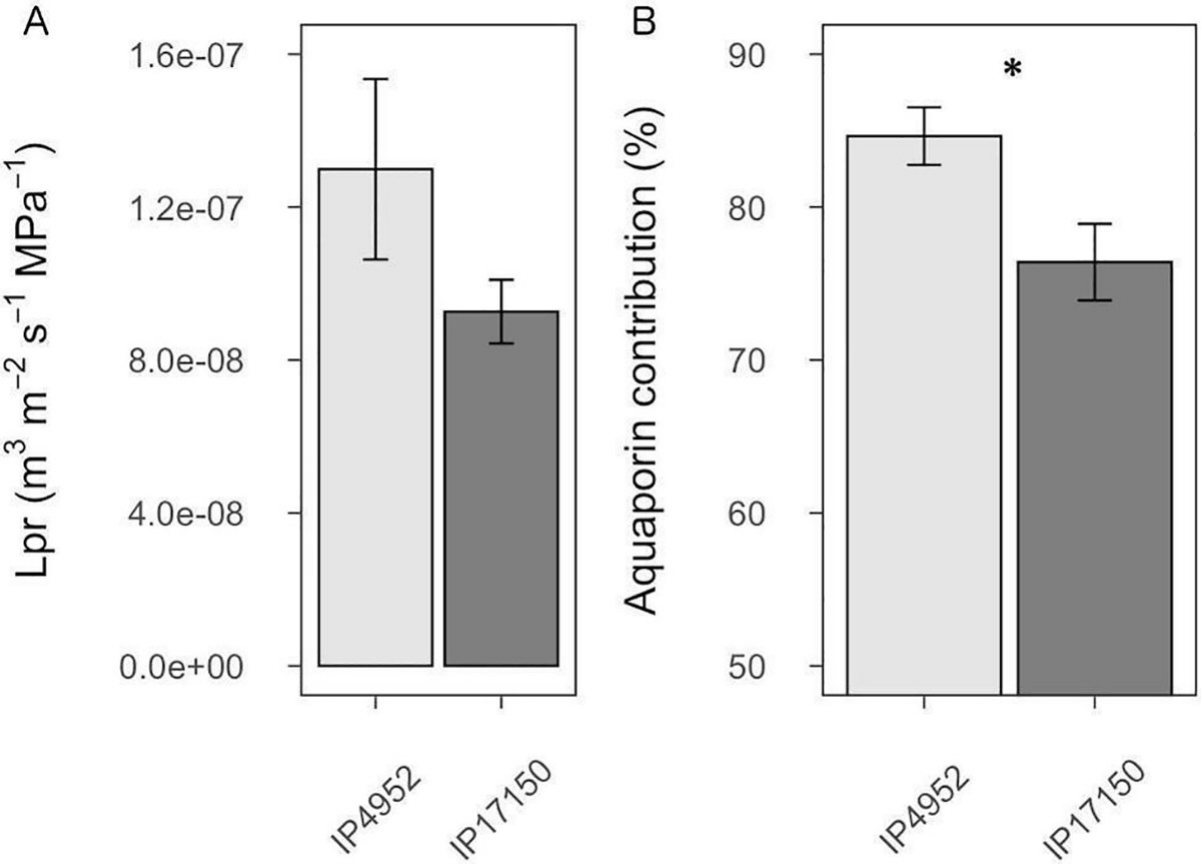
Root hydraulic conductivity and aquaporin contribution in roots of IP4952 and IP17150. Root hydraulic conductivity (Lpr) was measured in plants grown in hydroponic conditions between 9AM to 12PM with or without 2mM azide. (A) Lpr values were measured without azide. (B) Aquaporin contribution to Lpr shown as relative difference between Lpr of azide treated and non-azide treated plants. Bars represent mean values ± se of n = 10–15 plants. *: *p* < 0.05.

In order to determine if AQP function and root radial water flow could be associated with water use in pearl millet, we measured root hydraulic conductivity (Lpr) in IP4952 and IP17150, previously described as low and high water use efficiency lines, respectively. These lines did not show significant differences in root diameter and root surface area at the time of Lpr measurement, although root length was significantly higher in IP17150 as compared to IP4952 (p < 0.05; S5 Table). No significant differences in L_0_ and Lpr (*p* = 0.148) were observed between IP4952 and IP17150 (Fig 1 and S6 Table). In both IP4952 and IP17150, treatment with azide, an inhibitor of AQP activity, led to significant L_0_ and Lpr reduction (*p* < 0.001; S6 Table). Significant differences in L_0_ were observed between lines in the presence of azide (S6 Table), indicating genotypic differences in anatomical traits. This effect of azide application on Lpr was mostly reversible after treating the same roots with azide-free solution (S1 Fig). AQP contribution to Lpr was significantly higher in IP4952 (low water use efficiency) as compared to IP17150 (high water use efficiency; 84.64 ± 1.98% versus 76.40 ± 2.61%, *p* < 0.05; S6 Table). Therefore, Lpr inhibition by azide indicates that AQP could contribute more than 75% to Lpr in pearl millet.

### Aquaporin identification and annotation

To gain insight on the AQP isoforms contributing to Lpr in pearl millet, we characterized AQP gene families in the pearl millet genome. We blasted 772 AQP protein sequences identified in 19 different species on the pearl millet reference genome (chromosome assembly and scaffolds) [44]. A total of 7005 sequences with bit score >100, representing 50 specific hits were identified (S1 Table). Forty-seven of the hits fell within previously annotated genes, one fell in a non-annotated part of the genome on chromosome 5, and two in non-assembled parts of the genome (scaffold763 and scaffold8428).

Manual *de novo* annotation of the 50 putative AQP genes allowed the identification of eight genes with no start or with early stop codons in the first exon that were classified as pseudo-genes (S2 Table). Nine genes did not encode AQP isoforms but rather zinc-finger protein/LRR receptor-like serine-threonine protein kinase families or DEAD-like helicase-N protein superfamilies. The absence of AQP signature domains (NPA and Ar/R motifs) in their protein sequence confirmed their non-affiliation to the AQP family. A number of genes showed an excessive number of exons or longer first exon and were re-annotated on the basis of alignment with transcriptome sequences or with close protein homologs (Uniprot/Swiss-Prot blast results) and presence of AQP isoform conserved domains. In addition, ten genes showing missing sequences in coding regions were newly assembled and annotated after sequencing. Overall, 33 *Pennisetum glaucum* AQP (PgAQP) genes were identified in the pearl millet genome, sixteen of which were annotated *de novo* (Table 1).

**Table 1:** Description and distribution of pearl millet aquaporin genes.

ID	Gene	Pgl_Glean ID	Gene length (bp)	Transcript length (bp)	Protein length (aa)	Protein MW (kD)	Chr	Start	End
**Plasma membrane intrinsic proteins (PIP)**									
1	*PgPIP1-1*	*Pgl_GLEAN_10001520*	3097	867	288	30.70	3	5721622	5724718
2	*PgPIP1-3*	*Pgl_GLEAN_10010809*	1601	867	288	30.76	3	274573998	274575598
3	*PgPIP1-4*	*Pgl_GLEAN_10005724*	992	900	298	31.38	2	104247932	104248932
4	*PgPIP2-1*	*Pgl_GLEAN_10028064*	2920	873	290	30.35	3	12453415	12456334
5	*PgPIP2-2*	*Pgl_GLEAN_10028876*	2830	867	288	30.12	3	45603209	45606038
6	*PgPIP2-3*	*Pgl_GLEAN_10035675*	1903	873	290	30.39	3	257669631	257671533
7	*PgPIP2-5*	*Pgl_GLEAN_10028056*	1062	834	277	28.93	3	12167791	12168852
8	*PgPIP2-6*	*Pgl_GLEAN_10028055*	1053	861	286	29.94	3	12156953	12158005
9	*PgPIP2-7*	*Pgl_GLEAN_10010255*	1170	861	286	29.79	Scaffold763	240584	241753
10	*PgPIP2-8*	*Pgl_GLEAN_10009812*	837	837	278	29.17	2	64966663	64967453
**Tonoplast intrinsic proteins (TIP)**									
11	*PgTIP1-1*	*Pgl_GLEAN_10002147*	1499	750	249	25.72	5	153303973	153305471
13	*PgTIP2-1*	*Pgl_GLEAN_10000631*	844	744	247	24.88	2	30657555	30658398
16	*PgTIP2-2*	*Pgl_GLEAN_10030617*	933	747	248	25.03	3	100540239	100541171
12	*PgTIP2-3*	*Pgl_GLEAN_10009584*	851	750	249	25.06	3	33622445	33623295
15	*PgTIP3-1*	*Pgl_GLEAN_10028702*	911	801	266	27.41	2	44624536	44625481
14	*PgTIP4-1*	*Pgl_GLEAN_10002901*	2557	738	245	25.58	1	263875434	263878070
18	*PgTIP4-2*	*Pgl_GLEAN_10003219*	1455	744	247	25.15	3	148813464	148814777
17	*PgTIP4-3*	*Pgl_GLEAN_10003218*	844	747	248	25.10	3	148807632	148808412
19	*PgTIP5-1*	*Pgl_GLEAN_10033583*	1087	813	270	26.69	3	272599938	272601028
**Noduline-26 like intrinsic proteins (NIP)**									
20	*PgNIP1-1*	*Pgl_GLEAN_10012175*	2316	846	281	29.52	2	195368171	195370486
21	*PgNIP1-2*	*Pgl_GLEAN_10028618*	2560	846	281	29.53	1	261387113	261389753
22	*PgNIP1-4*	*Pgl_GLEAN_10028339*	1137	837	278	29.36	3	145385690	145386826
23	*PgNIP2-1*	*Pgl_GLEAN_10018521*	3364	891	296	31.82	3	14105646	14109009
24	*PgNIP2-2*	*Pgl_GLEAN_10019286*	3821	894	297	31.57	2	103033018	103036838
25	*PgNIP3-1*	*Pgl_GLEAN_10034621*	3855	909	302	31.43	2	40497062	40500916
26	*PgNIP3-2*	*Pgl_GLEAN_10030882*	1151	846	281	29.60	4	55609410	55610656
27	*PgNIP3-3*	*Pgl_GLEAN_10030883*	1087	780	259	27.05	4	55648949	55650374
28	*PgNIP3-4*	*Pgl_GLEAN_10030881*	1258	750	249	25.15	4	55573901	55575272
29	*PgNIP3-5*	*Pgl_GLEAN_10030872*	934	837	278	27.79	4	55508562	55509419
30	*PgNIP4-1*	*Pgl_GLEAN_10012100*	1319	921	306	31.52	6	110090448	110091766
**Small intrinsic proteins (SIP)**									
31	*PgSIP1-1*	*Pgl_GLEAN_10003744*	2818	726	241	25.32	1	175152169	175154986
32	*PgSIP1-2*	*Pgl_GLEAN_10014008*	3375	759	252	25.91	4	93394125	93397493
33	*PgSIP2-1*	*Pgl_GLEAN_10026167*	1899	759	252	27.10	5	126489209	126491163

*PgPIP2-7* is located on scaffold763, which was not assembled to the pearl millet genome. bp: base pairs; aa: amino-acids; MW: molecular weight; kD: kilo Dalton; Chr: chromosome; Start: position of the ATG; End: position of the stop codon.

### Pearl millet aquaporins phylogenic analysis

**Fig 2:**
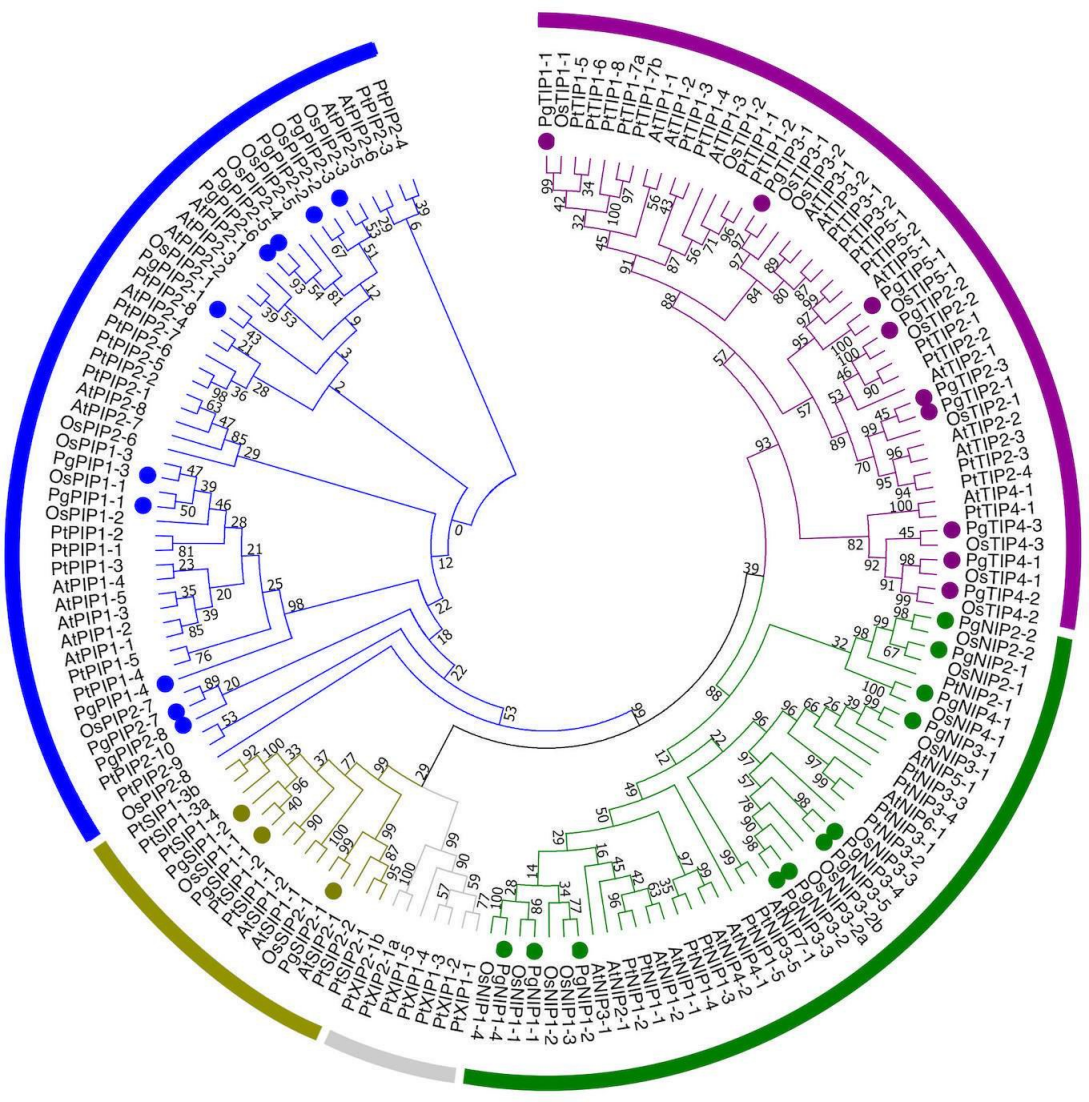
Phylogenetic relationship among aquaporins isoforms from pearl millet, Arabidopsis, rice and poplar. A phylogenetic tree was generated using the Maximum Likelihood method with 1000 reiterations in MEGA7. Bootstrap values above 50% are represented. The PIP, TIP, SIP, NIP and XIP family clades are represented by blue, grey, purple, green and red, respectively. Pearl millet sequences are indicated by colored dots.

To classify the PgAQP into families and name them, a phylogenetic tree was built using the PgAQP protein sequences along with protein sequences from *Arabidopsis thaliana*, *Oryza sativa* and *Populus tremula* (Fig 2). The PgAQP were named according to their grouping into families (PIP, TIP, SIP or NIP) and close homologs (Table 1). Ten isoforms showed homologies to the PIP family with three isoforms falling in the PIP1 sub-family (PgPIP1-1, PgPIP1-3 and PgPIP1-4) and seven isoforms falling in the PIP2 sub-family (PgPIP2-1, PgPIP2-2, PgPIP2-3, PgPIP2-5, PgPIP2-6, PgPIP2-7 and PIP2-8). Nine isoforms from the *P*. *glaucum* TIP family (PgTIP), eleven isoforms from the *P*. *glaucum* NIP family (PgNIP) and three isoforms from the *P*. *glaucum* SIP family (PgSIP) were further identified. No isoforms from the XIP family were identified in pearl millet. This analysis further confirmed the classification of PgPIP1-1, PgPIP2-1, PgPIP2-3, PgPIP2-6, PgTIP1-1 and PgTIP2-2 cloned by [31]. However, PgPIP1-2 from [31] was renamed as PgPIP1-3 in this study.

**Fig 3:**
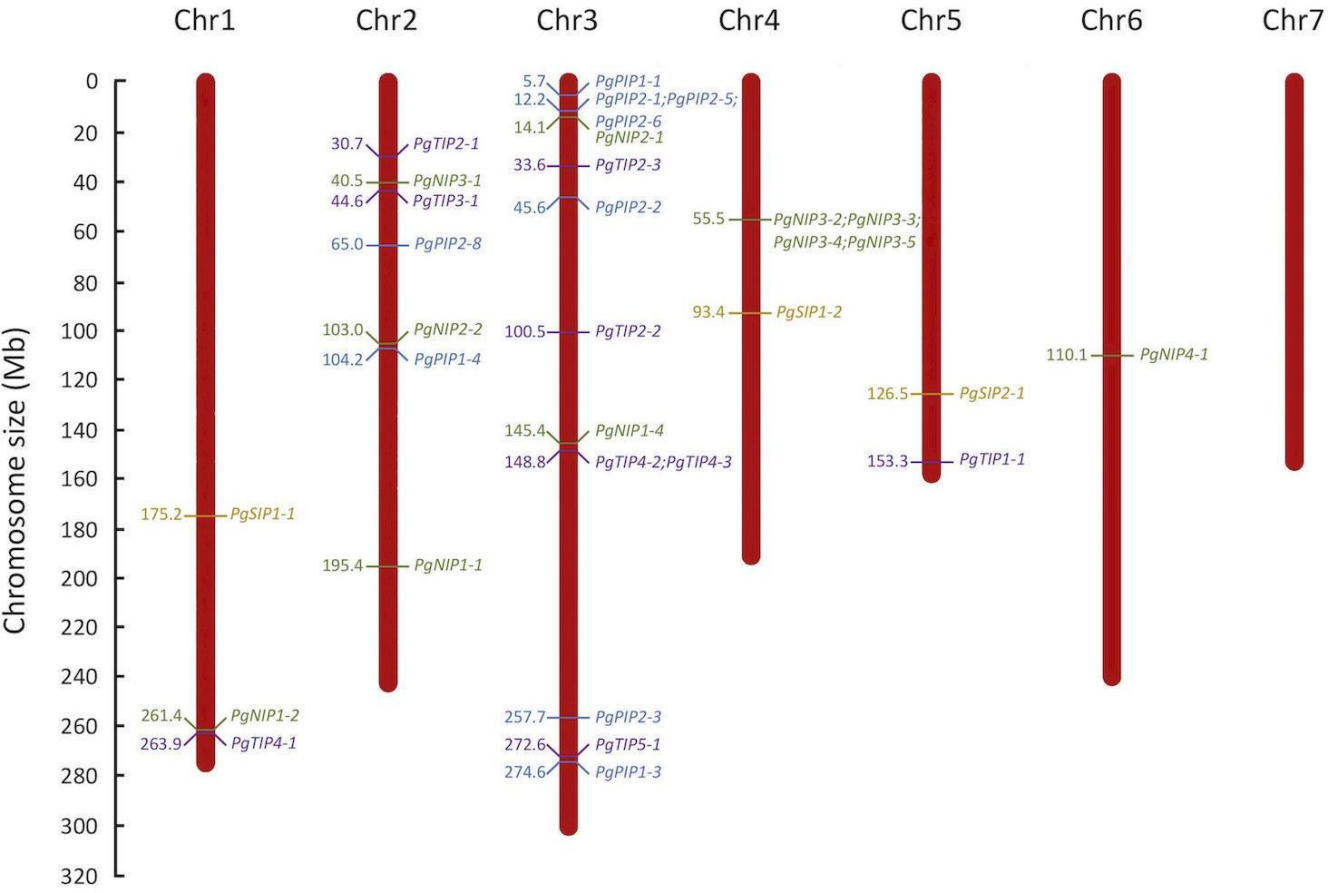
Distribution of aquaporin genes in the pearl millet genome. The seven chromosomes (Chr) of the pearl millet genome are represented according to their size in megabase (Mb). Positions of PIP, TIP, SIP and NIP genes are represented in blue, purple, orange and green, respectively. *PgPIP2-7*, which is localized on scaffold763, is not represented.

Most PgAQP genes were localized on chromosome 3 and none were localized on chromosome 7 (Fig 3). Two PgAQP hot-spots were observed, one in a region of 11899bp on chromosome 3 containing *PgPIP2-1*, *PgPIP2-5* and *PgPIP2-6* and the other in a region of 141812bp on chromosome 4 containing *PgNIP3-2*, *PgNIP3-3*, *PgNIP3-4* and *PgNIP3-5*.

### Aquaporin gene structure in pearl millet

PgAQP genes showed large variation in gene length (ranging from 837bp for *PgPIP2-8* to 3855bp for *PgNIP3;1*; Table 1). Transcript lengths were less variable and relatively conserved within families, with lengths of around 800-900bp for the PgPIP and PgNIP genes, 750-800bp for the PgTIP genes and 700-750bp for the PgSIP genes.

To further confirm the phylogenetic classification, gene structure of the PgAQP genes was analyzed (S2 Fig). PgAQP genes displayed between one (*PgPIP2-8*) and five exons (*PgNIP2-1*, *PgNIP2-2* and *PgNIP4-1*). Except for the NIP family, intron-exon organization was generally conserved within families with 5 of 9 PgPIP genes displaying 3 exons, 6 of 9 PgTIP genes displaying 2 exons and all PgSIP genes displaying 3 exons, supporting their phylogenetic distribution. Furthermore, *PgPIP2-5/PgPIP2-6* and *PgNIP3-2/PgNIP3-3* were found to be close homologs in the phylogenetic analysis (Fig 2), and were located near each other on the pearl millet genome (Fig 3). They showed similar gene and transcript length (Table 1) as well as gene structure (S2 Fig).

PgAQP coding regions encoded proteins with length varying between 250 to 300 amino acids, with molecular weight of around 30kD for the PgPIP and PgNIP and 25kD for the PgTIP and PgSIP isoforms (Table 1).

### Pearl millet AQP conserved domains

**Fig 4:**
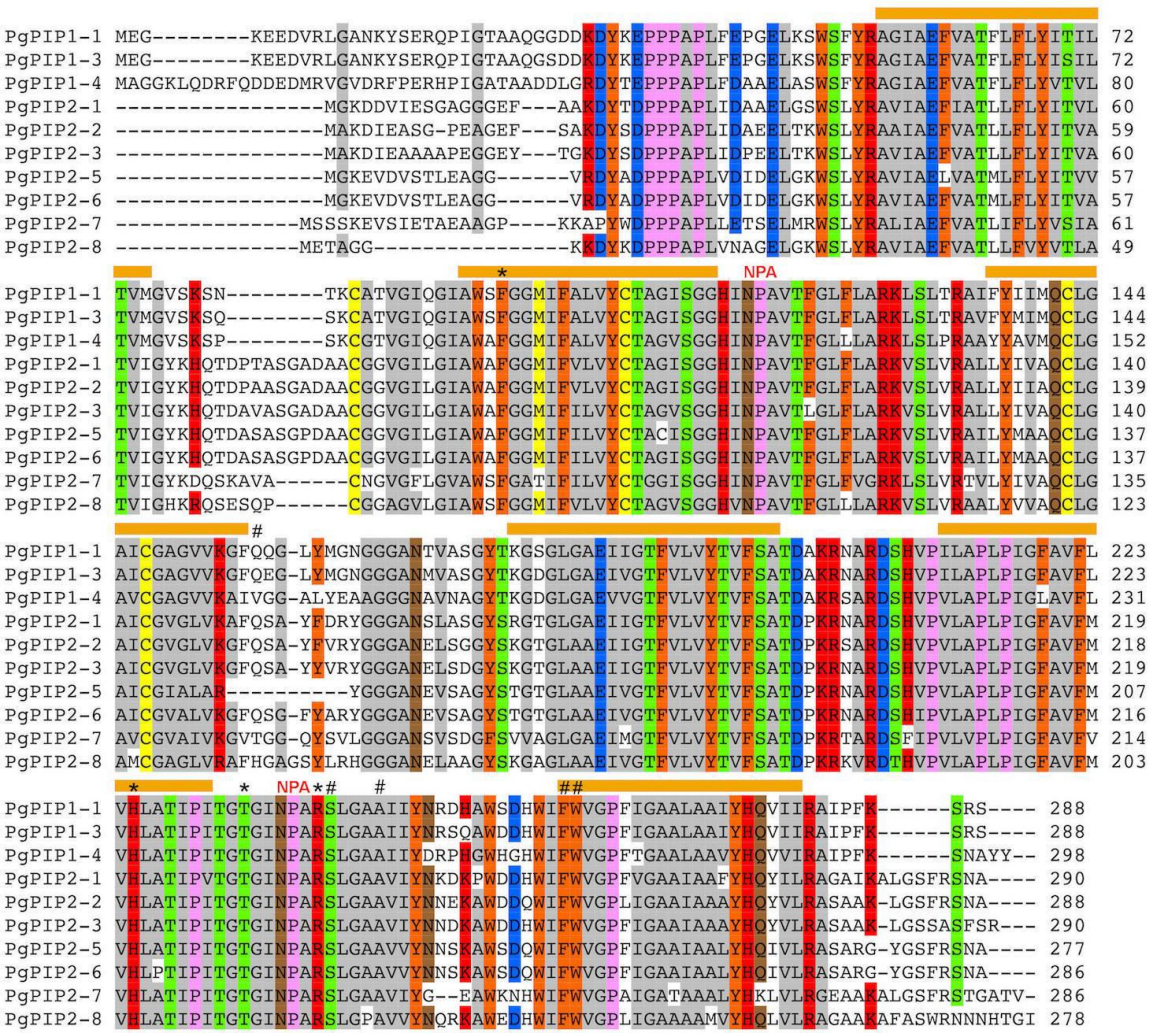
Conserved domains and membrane topology of the PIP isoforms from pearl millet. Alignment of the PIP isoforms were obtained using ClustalW in Mega7. Sequence identities and similarities (80%) are highlighted in colors. The transmembrane domains are represented by orange bars with the N-terminal and C-terminal ends of the protein located in the cytosol. NPA: Asparagine-Proline-Alanine motifs; *: Aromatic/Arginine selectivity filters. #: Froger’s residues.

Analysis of selectivity filters and transmembrane domains was performed to investigate polymorphisms that could impact substrate selectivity of the PgAQP. Analysis of conserved domains showed that all PgAQP isoforms belonged to the MIP super-family and displayed typical double NPA motifs (S7 Table and Table 2). Although some polymorphisms in NPA motifs were observed in some isoforms (particularly from the NIP family), subsequent amino-acids had the same chemical properties (generally neutral and non-polar; Table 2, Fig 4 *and* S3–S5 Figs). Ar/R selectivity filters and Froger’s residues were well conserved in the PgPIP isoforms, except for the Froger’s residue on position 1 (P1; Table 2, Fig 4). More polymorphisms were observed in these residues for the PgTIP, PgNIP and PgSIP isoforms although the ar/R residue on Loop E (R on LE2) and the Froger’s residues at positions 3 (A) and 4 (F/Y) were well conserved across all isoforms (Table 2).

**Table 2:** Amino-acids residues in conserved domains of pearl millet aquaporins isoforms.

Isoform	NPA (LB)	NPA (LE)	Ar/R selectivity filters				Froger’s residue				
			H2	H5	LE1	LE2	P1	P2	P3	P4	P5
**Plasma membrane intrinsic proteins (PIP)**											
PgPIP1-1	NPA	NPA	F	H	T	R	Q	S	A	F	W
PgPIP1-3	NPA	NPA	F	H	T	R	Q	S	A	F	W
PgPIP1-4	NPA	NPA	F	H	T	R	V	S	A	F	W
PgPIP2-1	NPA	NPA	F	H	T	R	Q	S	A	F	W
PgPIP2-2	NPA	NPA	F	H	T	R	Q	S	A	F	W
PgPIP2-3	NPA	NPA	F	H	T	R	Q	S	A	F	W
PgPIP2-5	NPA	NPA	F	H	T	R	-	S	A	F	W
PgPIP2-6	NPA	NPA	F	H	T	R	Q	S	A	F	W
PgPIP2-7	NPA	NPA	F	H	T	R	T	S	A	F	W
PgPIP2-8	NPA	NPA	F	H	T	R	H	S	A	F	W
**Tonoplast intrinsic proteins (TIP)**											
PgTIP1-1	NPA	NPA	H	I	A	V	T	S	A	Y	W
PgTIP2-1	NPA	TPA	H	I	G	R	T	S	A	Y	W
PgTIP2-2	NPA	NPA	H	I	G	R	T	S	A	Y	W
PgTIP2-3	NPA	NPA	H	I	G	R	T	S	A	Y	W
PgTIP3-1	NPA	NPA	H	V	A	R	T	V	A	Y	W
PgTIP4-1	NPS	NPA	N	S	A	R	T	S	A	Y	W
PgTIP4-2	NPA	NPA	Q	S	A	R	T	S	A	Y	W
PgTIP4-3	NPA	NPA	H	I	A	H	T	S	A	Y	W
PgTIP5-1	NPA	NPA	Q	V	A	R	R	S	A	Y	W
**Noduline-26 like intrinsic proteins (NIP)**											
PgNIP1-1	NPA	NPA	W	V	A	R	F	T	A	Y	V
PgNIP1-2	NPA	NPA	W	V	A	R	F	T	A	Y	F
PgNIP1-4	NPA	NPV	W	A	A	R	F	S	A	Y	I
PgNIP2-1	NPA	NPA	G	S	G	R	L	T	A	Y	F
PgNIP2-2	NPA	NPA	G	S	G	R	L	T	A	Y	F
PgNIP3-1	NPS	NPV	A	I	G	R	F	T	A	Y	L
PgNIP3-2	NPA	NPA	A	A	A	R	Y	T	A	Y	M
PgNIP3-3	NPA	NPA	A	A	A	R	Y	T	A	Y	M
PgNIP3-4	NPA	NPA	A	A	A	R	Y	T	A	Y	M
PgNIP3-5	NPA	NPA	A	A	A	R	Y	T	A	Y	M
PgNIP4-1	NPA	NPI	M	G	G	R	M	T	A	Y	L
**Small intrinsic proteins (SIP)**											
PgSIP1-1	NPT	NPA	V	V	P	N	M	A	A	Y	W
PgSIP1-2	NPT	NPA	L	I	P	N	M	A	A	Y	W
PgSIP2-1	NPL	NPA	S	H	G	S	F	A	A	Y	W

The two NPA (Asparagine-Proline-Alanine) motifs are located on loop B (LB) and loop E (LE). Aromatic/Arginine motifs (Ar/R) are located on helix 2 (H2), helix 5 (H5), and loop E (LE1 and LE2). P1-5 designed the five Froger’s position.

Transmembrane domain analyses using three different types of prediction software suggested a high probability that all identified PgAQP possess six transmembrane domains with cytoplasmic N-terminal and C-terminal ends, as is typically observed for AQP (S8 Table). Predictions of 3-dimensional geometric structure and pore morphology suggested a continuous pore that runs longitudinally across both sides of the membranes for all PgPIP (S6 Fig). Two constrictions in the pore center were typically observed at both extremities, illustrating the two constraints caused by the NPA motifs.

### Aquaporin expression profiling in pearl millet

**Fig 5:**
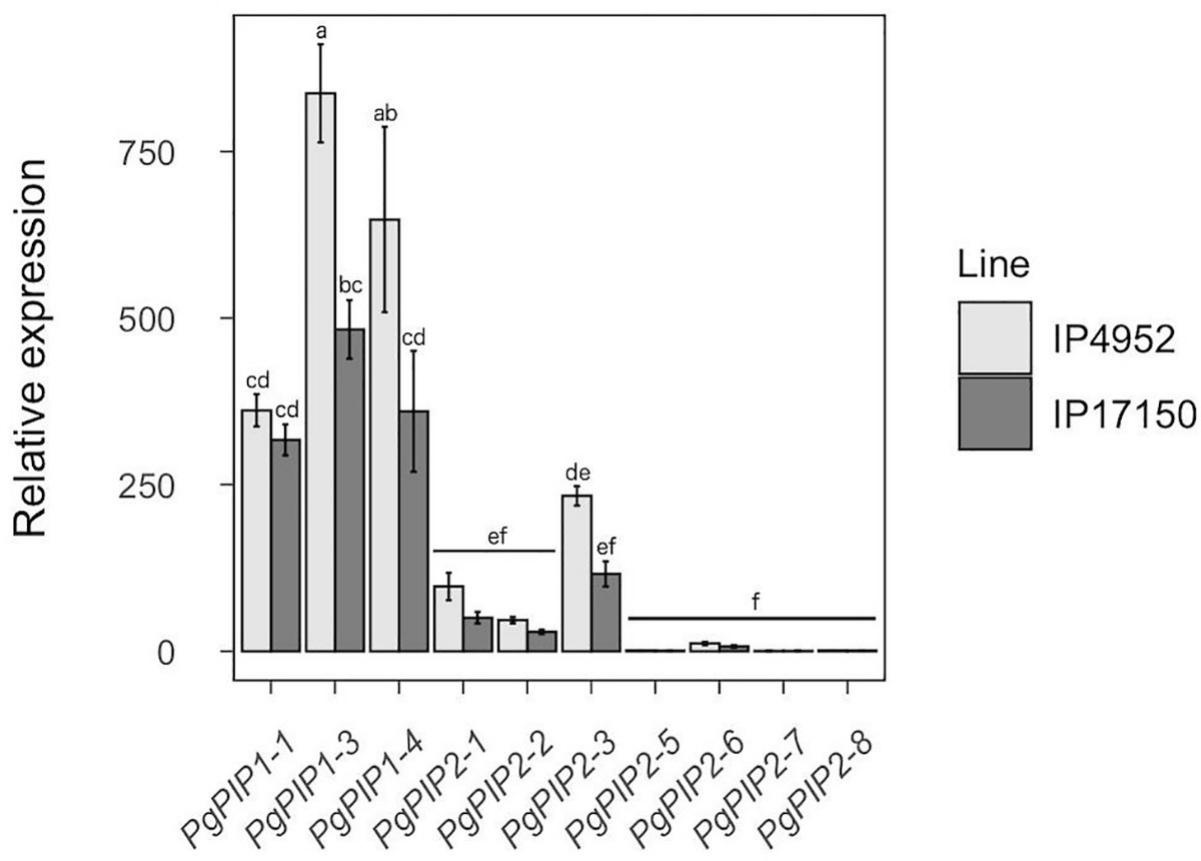
Relative expression of PIP genes in roots of IP4952 and IP17150. Transcript abundance of each PIP genes were measured between 9AM and 12PM on plants grown in hydroponic conditions and normalized to the expression of *PgPIP2-5* in IP4952. Bars show mean values ± se of n = 6–8 biological replicates, each with three technical replicates. Letters indicate significance difference among groups.

PIP isoforms are thought to play major roles in root hydraulic conductivity because of their localization at the plasma membrane [8]. To infer PgPIP isoforms putative importance to Lpr in pearl millet, we analyzed PgPIP gene expression pattern in roots of IP4952 and IP17150 using quantitative PCR. PgPIP genes were generally more expressed in IP4952 as compared to IP17150 (Fig 5). In both lines, *PgPIP1-1*, *PgPIP1-3*, *PgPIP1-4*, and *PgPIP2-3* were most expressed while *PgPIP2-5*, *PgPIP2-6*, *PgPIP2-7* and *PgPIP2-8* were less expressed. *PgPIP1-3* and *PgPIP1-4* were the only genes significantly differently expressed between both lines.

Expression of PgPIP in shoots (leaves and inflorescence) were retrieved from [50]. Transcriptomic analyses from ten pearl millet varieties suggest that *PgPIP1-4* and *PgPIP2-3*, two of the most expressed genes in roots, are expressed at low levels in shoots (S7 Fig). Conversely, *PgPIP1-1* and *PgPIP1-3* are highly expressed in roots and shoots.

## Discussion

Investigation of the role of AQP in root water transport in pearl millet, a heat and drought-adapted crop, suggest that PgAQP are major contributors to root water flow. Root hydraulic conductivity (Lpr) varied around 1E-07 m^3^ m^-2^ s^-1^ MPa^-1^, within the previously report range of other plants [55]. Treatment of roots with a common AQP inhibitor (azide) suggested that AQP contribute up to 84% of Lpr in pearl millet (S6 Table). This figure is higher than what has been observed in Arabidopsis (57 to 64%) [56] and rice (42 to 79%) [57]. However, complete Lpr recovery following inhibition by azide (S1 Fig) suggests AQP contribution was not over-estimated due to off-target effects of azide.

The AQP gene family of the pearl millet genome was characterized and thirty-three putatively functional AQP isoforms (based on conserved domains and protein topology) were identified, belonging to the PIP (10), TIP (9), SIP (3) and NIP (11) families. No XIP were identified, which confirm the absence of isoforms from this family in the monocotyledon clade [8]. The number of AQP identified in pearl millet is similar to what has been observed in Arabidopsis (35) [58], rice (33) [23] and maize (31) [20]. Interestingly, AQP genes were over-represented on Chromosome 3 with fourteen genes (Fig 3). The proximal localization of *PgPIP2-5* and *PgPIP2-6* in Chromosome 3, their phylogenetical relatedness and similar gene structure (Fig 3 and S2 Fig), suggest possible tandem duplication events in this region [59]. Similarly, *PgNIP3-2* and *PgNIP3-3*, located on Chromosome 4 may be the result of duplication events. Furthermore, *PgPIP2-7* was identified in scaffold763 suggesting that genes may be missing on parts of the pearl millet genome assembly.

The selectivity of an AQP is defined by the three dimensional structure of the amino-acids constituting its pore. The NPA motifs on loops B and E contribute to the dipole moment of the α-helices and prevent proton permeation [60, 61]. These motifs were strictly conserved in PgPIP but showed some polymorphisms for other isoforms (Table 2). However, these substitutions did not drastically change the positive electrostatic potential at the NPA motifs, suggesting that proton exclusion from AQP pores is conserved in pearl millet. Furthermore, the Isoleucine (I) preceding the Froger’s residue P4 and P5 at the end of Loop E, shown to be essential for CO_2_ transport in PIP [62], is conserved in PgPIP (Fig 4).

The ar/R motifs, composed of four amino-acids, restrict extracytosolic access to the pore and constitute the main selectivity filter. Modelling approaches based on ar/R signatures were used to predict permeability of plant AQP [12, 13]. For instance, the F-H-T-R signature observed in the PgPIP (Table 2), which seems strictly conserved across PIP from different species, has been associated with water and H_2_O_2_ permeability [14, 26, 27, 63]. Furthermore, PgPIP1-3 carries the same ar/R motif and similar Froger’s residues as OsPIP1-3, which has been recently shown to transport nitrate (NO_3_^−^) [19]. The H-I-G-R signature observed in PgTIP2-1, PgTIP2-2 and PgTIP2-3, that is conserved in TIP2 from Arabidopsis, maize and rice, supposedly allows permeability to water, NH_3_, urea and H_2_O_2_. The H-I-A-V signature, observed in PgTIP1 (PgTIP1-1), may allow permeability to NH_3,_ urea and H_2_O_2_ but restrict water permeability [64–66]. In PgNIP, the W-V-A-R signature have been associated with water, NH_3_ and H_2_O_2_ permeability, while the A-I/A-G/A-R signatures were associated with restricted water and NH_3_ permeability. Interestingly, PgNIP2-1 and PgNIP2-2 showed ar/R signatures (G-S-G-R) similar to OsNIP2-1, which is permeable to silicon [18], and also possesses precisely 108 amino-acids between the two NPA motifs, which is assumed to be essential for silicon permeability [67].

Apart from the selectivity filters, a numbers of residues involved in PIP molecular gating mechanisms or subcellular localization were also conserved in PgPIP [8]. For instance, the Histidine (H) on Loop D (H199 in AtPIP2-1), which can be protonated to stabilize the pore in a closed conformation, was conserved in most PgPIP [61, 63, 68]. Furthermore, two Serine residues (S), located on Loop B and C-ter (S121 and S280 in AtPIP2-1) that would favor an open-pore conformation when phosphorylated, were conserved in PgPIP2 [61, 69]. Another Serine residue located on C-ter (S283 in AtPIP2-1), involved in PIP trafficking, was conserved in the PgPIP, with the exception of PgPIP2-7 [70]. Other residues located on the N-ter (Lysine 3 and Glutamate 6 in AtPIP2-1), subjected to methylation and involved in PIP trafficking were also conserved in most PgPIP [71]. Some of these post-translational modification mechanisms can affect aquaporin function in response to particular abiotic or nutritional stimuli and may well be conserved in pearl millet [63, 70, 72, 73].

AQP in plants are expressed in roots and shoots, including inflorescence and pollen. Some PgPIP genes show tissue-specific expression with *PgPIP1-4* and *PgPIP2-3* having higher expression levels in roots, while *PgPIP2-1* is expressed more in shoots and *PgPIP1-1* and *PgPIP1-3* are expressed in both roots and shoots (Fig 5 and S7 Fig). It has been shown that PIP isoforms agglomerate as tetramers in the plasma membrane, each monomer forming functional units. Functional studies as well as protein-protein interactions studies suggest that PIP tetramers can be formed of heteromers of PIP1 and PIP2 with distinct functional properties depending on the isoform combination [74–77]. Based on PgPIP gene expression in pearl millet, PgPIP1-1, PgPIP1-3 and PgPIP1-4 might interact with PgPIP2-3 to form heteromers in roots while PgPIP1-1, PgPIP1-3 and PgPIP2-1 might form different combinations of heteromers in shoots.

Intraspecific diversity in AQP isoform expression has been observed in rice and Arabidopsis [56, 57, 78, 79]. In our study, diversity in expression patterns of PgAQP genes were observed between two pearl millet inbred lines contrasting for water use strategy (Fig 5). IP4952 showed significantly greater AQP contribution to Lpr as compared to IP17150 and also showed significantly higher *PgPIP1-3* and *PgPIP1-4* gene expression. These results suggest that differences in expression of these AQP genes may reflect differences in AQP contribution to Lpr in pearl millet. These observations are in line with results from [35] showing that the expression of *VvPIP1-1* is associated with root hydraulics and response to water stress in two isohydric and anisohydric grapevine (*Vitis vinifera*) cultivars. Transpiration response to high VPD in four pearl millet recombinant inbred lines has been linked to PgPIP gene expression in roots [31]. The same work suggested that a down-regulation of PgPIP genes under high VPD induced reduction in transpiration and water savings. Our results show lower expression of *PgPIP1-3* and *PgPIP1-4* in IP17150 as well as a reduced role of AQP in Lpr in IP17150, the line with higher water use efficiency. These findings support the observations of [31]. More recently, overexpression of rice OsPIP1-3 enhanced water use efficiency in transgenic tobacco plants [19]. However, this response may well be linked to higher photosynthesis rates due to improved nitrate uptake and transport rather than hydraulics. Overall, expression profiling suggests that APQ may have different physiological functions across the pearl millet plant and contribute to its response to the environment. However, expression alone is certainly not fully representative of AQP function due to various types of post-translational regulations affecting AQP function [8]. Further investigations are needed to better understand the links between reduced transpiration under high VPD, improved water use efficiency and AQP function in roots.

Pearl millet is a drought-adapted crop that will play a major role in the adaptation of agriculture to future climates in arid and semi-arid regions of Africa and India. Here, we provide a comprehensive view of the AQP genes and isoforms present in pearl millet as well as their contribution in root radial water transport. Our results suggest that differential expression of PgPIP1-3 and PgPIP1-4 may be associated with different water use strategies. Therefore, our study supports a potential role of these two AQP isoforms in regulating pearl millet hydraulics and potentially adaptation to challenging environmental conditions.

## Supporting information

S1 Table High scoring pairs with highest bit score at the fifty hot-spots. (PDF)

S2 Table Functional annotation of the pearl millet genomic regions corresponding to hotspots of high scoring pairs. (PDF)

S3 Table Primers used for genomic DNA (gDNA) or complementary DNA (cDNA) amplification of aquaporins showing missing sequence. (PDF)

S4 Table Primers used for quantitative RT-PCR. (PDF)

S5 Table Root architectural traits in IP4952 and IP17150. (PDF)

S6 Table Root conductance (L_0_), root hydraulic conductivity (Lpr) and aquaporin (AQP) contribution in IP4952 and IP17150. (PDF)

S7 Table Analysis of aquaporin conserved domains in pearl millet. (PDF)

S8 Table Transmembrane domain analysis of aquaporins in pearl millet. (PDF)

S1 Fig Reversion of azide-induced root hydraulic conductivity inhibition. (PDF)

S2 Fig Structure of pearl millet aquaporins genes. (PDF)

S3 Fig Conserved domains and membrane topology of the TIP isoforms from pearl millet. (PDF)

S4 Fig Conserved domains and membrane topology of the NIP isoforms from pearl millet. (PDF)

S5 Fig Conserved domains and membrane topology of the SIP isoforms from pearl millet. (PDF)

S6 Fig Cavity features of PgPIP isoforms. (PDF)

S7 Fig Expression pattern of PgPIP isoforms in shoots of pearl millet. (PDF)
