# Supplementary material for: Aquaporins are main contributors to root hydraulic conductivity in pearl millet [Pennisetum glaucum (L) R. Br.]
Source: PLoS One. 2020 Oct 1;15(10):e0233481. doi: 10.1371/journal.pone.0233481 (PMC7529256; doi:10.1371/journal.pone.0233481)
Supplement: S1 Table — (PDF) [file pone.0233481.s001.pdf]

S1 Table. High scoring pairs with highest bit score at the fifty hot-spots.

Hot-spot ID	Query	Blast Hit	Chromosome/ Scaffold	% identity	Alignment length	Mismatch	e-value	Bit score
1	SiPIP1-2	*Pgl_GLEAN_10001520*	3	81.944	144	19	1.09E-66	230
2	PgPIP1-2	*Pgl_GLEAN_10010809*	3	82.171	258	4	8.16E-137	413
3	ZmPIP1-5	*Pgl_GLEAN_10005724*	2	89.219	269	23	3.23E-150	470
4	PgPIP2-1	*Pgl_GLEAN_10028064*	3	100	206	0	1.75E-129	410
5	SiPIP2-4	*Pgl_GLEAN_10028876*	3	97.573	206	5	3.32E-127	404
6	SiPIP2-3	*Pgl_GLEAN_10035675*	3	98.068	207	4	9.23E-129	408
7	PgPIP2-6	*Pgl_GLEAN_10028056*	3	96.569	204	7	1.33E-124	396
8	PgPIP2-6	*Pgl_GLEAN_10028055*	3	99.51	204	1	1.28E-128	407
9	SiPIP2-7	*Pgl_GLEAN_10009812*	2	92.5	160	12	9.63E-143	300
10	SiPIP2-8	*Pgl_GLEAN_10010255*	Scaffold763	68.831	385	17	1.83E-156	485
11	ZmPIP2-1	Not annotated	5	82.278	79	14	2.30E-33	134
12	SiTIP1-2	*Pgl_GLEAN_10002147*	5	98.039	204	4	1.58E-102	331
13	PvTIP2-2	*Pgl_GLEAN_10000631*	2	96.117	206	7	1.95E-124	387
14	SiTIP2-4	*Pgl_GLEAN_10030617*	3	99.18	122	1	2.05E-115	241
15	PvTIP2-1	*Pgl_GLEAN_10009584*	3	90.732	205	18	8.59E-116	363
16	PvTIP3-1	*Pgl_GLEAN_10028702*	2	87.023	131	14	1.04E-61	214
17	SiTIP4-4	*Pgl_GLEAN_10002901*	1	95.122	123	6	1.83E-72	245
18	SiTIP4-2	*Pgl_GLEAN_10003219*	3	61	100	24	3.94E-22	106
19	BdTIP4-2	*Pgl_GLEAN_10003218*	3	69.388	98	29	2.30E-33	135
20	BdTIP5-1	*Pgl_GLEAN_10033583*	3	60.284	141	42	1.27E-49	146
21	ZmSIP1-6	*Pgl_GLEAN_10002144*	Scaffold8428	88.889	81	9	1.52E-36	152
22	SiNIP1-1	*Pgl_GLEAN_10012175*	2	95.172	145	7	3.99E-87	288
23	SiNIP1-2	*Pgl_GLEAN_10028618*	1	77.381	168	11	2.60E-73	248
24	SiNIP1-3	*Pgl_GLEAN_10028339*	3	88.235	136	14	3.38E-123	251
25	SiNIP2-3	*Pgl_GLEAN_10018521*	3	68.987	158	1	1.36E-57	204
26	SiNIP2-2	*Pgl_GLEAN_10019286*	2	98.78	82	1	1.01E-45	170
27	PvNIP3-10	*Pgl_GLEAN_10034621*	2	76.395	233	33	7.56E-111	318
28	SiNIP3-1	*Pgl_GLEAN_10030882*	4	76.503	183	37	3.03E-98	266
29	SiNIP3-1	*Pgl_GLEAN_10030883*	4	78.788	264	21	1.82E-128	370
30	SiNIP3-1	*Pgl_GLEAN_10030881*	4	57.312	253	67	1.23E-72	247
31	SbNIP3-2	*Pgl_GLEAN_10030872*	4	79.31	116	23	2.00E-42	160
32	SiNIP4-1	*Pgl_GLEAN_10012100*	6	75.581	172	11	9.31E-117	251
33	PvSIP1-2	*Pgl_GLEAN_10003744*	1	78.744	207	13	2.14E-82	273
34	BdSIP1-1	*Pgl_GLEAN_10014008*	4	84.337	83	11	3.16E-37	143
35	OsSIP2-1	*Pgl_GLEAN_10026167*	5	75	96	24	3.16E-38	146
36	SbPIP2-7	*Pgl_GLEAN_10014426*	7	80	85	15	9.67E-32	129
37	SiPIP2-2	*Pgl_GLEAN_10028060*	3	97.059	204	6	2.07E-124	395
38	SbPIP2-1	*Pgl_GLEAN_10028061*	3	69.492	118	1	2.13E-39	151
39	SbPIP2-7	*Pgl_GLEAN_10034735*	4	84.706	85	12	5.59E-36	141
40	SlTIP2-2	*Pgl_GLEAN_10003221*	3	60.369	217	75	3.64E-104	252
41	BdTIP4-2	*Pgl_GLEAN_10037924*	2	65.487	113	36	1.79E-37	147
42	SiNIP3-4	*Pgl_GLEAN_10030880*	4	93.388	121	8	3.56E-57	202
43	PvNiP1-3	*Pgl_GLEAN_10036237*	6	55.195	154	56	1.80E-31	129
44	ZmSIP1-6	*Pgl_GLEAN_10000628*	1	51.282	117	55	6.87E-28	127
45	ZmSIP1-3	*Pgl_GLEAN_10002205*	3	95	60	3	4.04E-27	121
46	ZmSIP1-6	*Pgl_GLEAN_10006441*	2	93.478	92	6	1.55E-47	189
47	ZmSIP1-2	*Pgl_GLEAN_10010441*	6	93.333	60	4	3.38E-28	124
48	ZmSIP1-6	*Pgl_GLEAN_10012193*	6	67.136	213	37	2.18E-79	286
49	ZmSIP1-6	*Pgl_GLEAN_10014461*	7	53.261	92	42	3.12E-23	112
50	ZmSIP1-6	*Pgl_GLEAN_10022843*	2	60.227	88	34	9.34E-25	117

772 aquaporin protein sequences from 19 plant species (queries) were blasted (tblastn) against the pearl millet genome (ASM217483v2) and the non-assembled scaffolds. An e-value of 10^{-5} was used as initial cut-off to identify high scoring pairs. High scoring pairs with bit score ≥100 allowed the identification of 50 hotspots of high scoring pairs which mostly fall into annotated genes.
