# Supplementary material for: Aquaporins are main contributors to root hydraulic conductivity in pearl millet [Pennisetum glaucum (L) R. Br.]
Source: PLoS One. 2020 Oct 1;15(10):e0233481. doi: 10.1371/journal.pone.0233481 (PMC7529256; doi:10.1371/journal.pone.0233481)
Supplement: S2 Table — (PDF) [file pone.0233481.s002.pdf]

S2 Table. Functional annotation of the pearl millet genomic regions corresponding to hotspots of High Scoring Pairs.

Hotspot ID	Isoform ID	Gene ID	KEGG IDs	Swiss-prot IDs	InterPro IDs	Manual annotation	Remarks
1	PgPIP1-1	*Pgl_GLEAN_10001520*	K09872	Q6EU94	IPR000425 IPR022357	No	-
2	PgPIP1-3	*Pgl_GLEAN_10010809*	K09872	Q6EU94	IPR000425 IPR022357	No	-
3	PgPIP1-4	*Pgl_GLEAN_10005724*	K09872	Q9ATN0	IPR000425 IPR022357	No	-
4	PgPIP2-1	*Pgl_GLEAN_10028064*	K09872	Q84RL7	IPR000425 IPR022357	No	-
5	PgPIP2-2	*Pgl_GLEAN_10028876*	K09872	Q9ATM6	IPR000425 IPR022357	No	-
6	PgPIP2-3	*Pgl_GLEAN_10035675*	K09872	Q9XF58	IPR000425 IPR022357	No	-
7	PgPIP2-5	*Pgl_GLEAN_10028056*	K09872	Q9ATM5	IPR000425 IPR022357	Yes	Longer exon 1 based on protein homology and transmembrane domains
8	PgPIP2-6	*Pgl_GLEAN_10028055*	K09872	Q9ATM5	IPR000425 IPR022357	No	-
9	PgPIP2-7	*Pgl_GLEAN_10010255*	K09872	Q651D5	IPR000425	No	-
10	PgPIP2-8	*Pgl_GLEAN_10009812*	K09872	Q8GRI8	IPR000425 IPR022357	Yes	Resequencing – new sequence added
11	-	*ZmPIP2-1* like PgPIP	K09872	Q84RL7	IPR000425	No	Pseudo-gene. No start codon and early stop
12	PgTIP1-1	*Pgl_GLEAN_10002147*	K09873	O64964	IPR000425 IPR022357	No	-
13	PgTIP2-1	*Pgl_GLEAN_10000631*	K09873	Q84RL6	IPR000425 IPR022357	No	-
14	PgTIP2-2	*Pgl_GLEAN_10030617*	K09873	Q5Z6F0	IPR000425 IPR022357	No	-
15	PgTIP2-3	*Pgl_GLEAN_10009584*	K09873	Q9ATL8	IPR000425 IPR022357	No	-
16	PgTIP3-1	*Pgl_GLEAN_10028702*	K09873	Q9ATL7	IPR000425	Yes	Resequencing – new sequence added sequence with fusion between exon2 and 3
17	PgTIP4-1	*Pgl_GLEAN_10002901*	K09873	Q9ATL5	IPR000425 IPR022357	Yes	Resequencing – New sequence added in exon 1
18	PgTIP4-2	*Pgl_GLEAN_10003219*	K09873	Q9ATL4	IPR000425 IPR022357	Yes	Resequencing – new sequence added in exon 2 with longer protein
19	PgTIP4-3	*Pgl_GLEAN_10003218*	K09873	Q9ATL3	IPR000425	Yes	Resequencing – new sequence added in exon 2 with longer protein
20	PgTIP5-1	*Pgl_GLEAN_10033583*	K09873	Q7XU31	IPR000425	Yes	Resequencing – with longer exon2 and exon 3
21	-	*Pgl_GLEAN_10002144*	K17679	Q6K7R9	-	No	DEAD-like helicase-N super family
22	PgNIP1-1	*Pgl_GLEAN_10012175*	K09874	Q9ATN4	IPR000425 IPR022357	No	-

23	PgNIP1-2	*Pgl_GLEAN_10028618*	K09874	Q0DK16	IPR000425 IPR022357	Yes	Resequencing – new sequence added with fusion of exon 2 and 3
24	PgNIP1-4	*Pgl_GLEAN_10028339*	K09874	Q5Z9E2	IPR000425	Yes	Longer exon1 including NPA motif on Loop B
25	PgNIP2-1	*Pgl_GLEAN_10018521*	K09874	Q19KC1	IPR000425	No	-
26	PgNIP2-2	*Pgl_GLEAN_10019286*	K09874	Q9ATN2	IPR000425 IPR022357	No	-
27	PgNIP3-1	*Pgl_GLEAN_10034621*	K09874	Q9ATN1	IPR000425 IPR022357	No	-
28	PgNIP3-2	*Pgl_GLEAN_10030882*	K09874	Q7EYH7	IPR000425 IPR022357	Yes	Shorter N-terminus based on protein homology
29	PgNIP3-3	*Pgl_GLEAN_10030883*	K09874	Q7EYH7	IPR000425 IPR022357	Yes	Shorter N-terminus and C-terminus based on protein homology and RNA-seq reads
30	PgNIP3-4	*Pgl_GLEAN_10030881*	K09874	Q7EYH7	IPR000425 IPR022357	Yes	Shorter N-terminus based on protein homology and transmembrane domains
31	PgNIP3-5	*Pgl_GLEAN_10030872*	K09874	Q7EYH7	IPR000425	Yes	Resequencing – Longer exon 2 based on added new sequence
32	PgNIP4-1	*Pgl_GLEAN_10012100*	K09874	Q9ASI1	IPR000225	Yes	9 exons in original annotation. Removal of first 5 exons and last exon.
33	PgSIP1-1	*Pgl_GLEAN_10003744*	K09875	Q9ATM2	IPR000425	No	-
35	PgSIP1-2	*Pgl_GLEAN_10014008*	K09875	Q9ATM3	IPR000425	Yes	Resequencing- New sequence added in exon 1
34	PgSIP2-1	*Pgl_GLEAN_10026167*	K09875	Q9SG80	IPR000425	Yes	21 exons in original annotation. Removal of first 19 exons. Resequencing with new sequence added on N-ter.
36	-	*Pgl_GLEAN_10014426*	K09872	Q84RL7	IPR000425	No	Pseudo-gene. Early stop
37	-	*Pgl_GLEAN_10028060*	K09872	Q9ATM5	IPR000425 IPR022357	No	Pseudo-gene. Early stop
38	-	*Pgl_GLEAN_10028061*	K09872	Q9ATM5	IPR000425	No	Pseudo-gene. Early stop
39	-	*Pgl_GLEAN_10034735*	K09872	Q84RL7	IPR000425	No	Pseudo-gene. Early stop
40	-	*Pgl_GLEAN_10003221*	K00784	Q80Y81	-	No	Zinc phosphodiesterase ELAC protein 2
41	-	*Pgl_GLEAN_10037924*	K09873	Q9ATL3	IPR000425	No	Pseudo-gene. Absence of conserved domain, short protein caused by a stop codon in exon 1.
42	-	*Pgl_GLEAN_10030880*	K09874	Q84S07	IPR000425	No	Pseudo-gene. Absence of

							conserved domain, short protein caused by a stop codon in exon 1.
43	-	*Pgl_GLEAN_10036237*	K09874	Q0JPT5	IPR000425	No	Pseudo-gene. Absence of conserved domain probably due to shorter protein caused by a stop codon in exon 2.
44	-	*Pgl_GLEAN_10000628*	K10144	O14099	IPR001841	No	Zinc-Finger protein
45	-	*Pgl_GLEAN_10002205*	K12870	Q6AYB3	IPR009360	No	Isy1-like splicing factor
46	-	*Pgl_GLEAN_10006441*	K13415	C0LGK4	IPR000719	No	LRR receptor-like serine/threonine-protein kinase
47	-	*Pgl_GLEAN_10010441*	K12861	Q949S9	IPR008409	No	pre-mRNA-splicing factor SPF27
48	-	*Pgl_GLEAN_10012193*	K10144	O14099	IPR001841	No	Ring finger and CHY zinc finger domain-containing protein 1
49	-	*Pgl_GLEAN_10014461*	K13415	C0LGK4	IPR000719	No	LRR receptor-like serine/threonine-protein kinase
50	-	*Pgl_GLEAN_10022843*	K13415	C0LGK4	IPR000719<br>IPR001611	No	LRR receptor-like serine/threonine-protein kinase

Forty-nine of the identified hot-spots corresponded to annotated genes (except for hot-spot 11). Functional characteristics of the encoded proteins were studied using KEGG, Uniprot/Swiss-Prot and Inter-Pro databases. First blast hit ID obtained at each database are presented. If the genomic sequence were subjected to manual annotation (yes), a description of the *de novo* annotation is provided (remarks).
