# Supplementary material for: Aquaporins are main contributors to root hydraulic conductivity in pearl millet [Pennisetum glaucum (L) R. Br.]
Source: PLoS One. 2020 Oct 1;15(10):e0233481. doi: 10.1371/journal.pone.0233481 (PMC7529256; doi:10.1371/journal.pone.0233481)
Supplement: S3 Table — (PDF) [file pone.0233481.s003.pdf]

**S3 Table. Primers used for genomic DNA (gDNA) or complementary DNA (cDNA) amplification of aquaporins showing missing sequence.**

| Gene | Sequence amplified | Forward (5’ -> 3’) | Reverse (5’ -> 3’) |
|---|---|---|---|
| *PgPIP2-5* | gDNA | CGGGAGTTGCTCTAAACCTG | TTCCCCATGGAATCAGAGAG |
| *PgPIP2-8* | gDNA | CGAGAACACGGTGTAGACGA | GTCACCTTCGGCCTCCTG |
| *PgTIP3-1* | gDNA | GGCGTAGTAGGCGTACATGA | CGTGGCCGTCAACATCTC |
| *PgTIP4-1* | cDNA | GAACAACAGAGCGAGGAACC | AGGATCACCTCCATCACCAC |
| *PgTIP4-2* | gDNA | GCCAGATCACCCTGTTCC | CAGCTGATGATGGTGTGCAG |
| *PgTIP4-3* | gDNA | ACAATGCACGCAGGCAAG | GCGTACACCGTGAAGAGCAG |
| *PgNIP1-2* | gDNA | CACCGTCCCGAAGAAGTG | CCGTCATGGTGATGGTCTACT |
| *PgNIP3-5* | gDNA | CTGGTCTTCATCGTGCTGTC | CGAAGAGGAAGACGAAGGTG |
| *PgSIP1-2* | cDNA | TGCAGTTTCCAAATTCCTGA | GCGAGCGTGTGCTTGTACT |
| *PgSIP2-1* | gDNA | CAATTCCAACGAGGGCTAAA | TAGACGAGCAGCTTCACCAG |
