# Supplementary material for: Aquaporins are main contributors to root hydraulic conductivity in pearl millet [Pennisetum glaucum (L) R. Br.]
Source: PLoS One. 2020 Oct 1;15(10):e0233481. doi: 10.1371/journal.pone.0233481 (PMC7529256; doi:10.1371/journal.pone.0233481)
Supplement: S4 Table — (PDF) [file pone.0233481.s004.pdf]

S4 Table. Primers used for quantitative RT-PCR.

Gene	Forward (5’ -> 3’)	Reverse (5’ -> 3’)	Primer efficiency (%)
PgPIP1-1	AGGTGATCATCAGGGCCATC	GGGAGACGAGGAATTGACAG	94
PgPIP1-3	CCTGGCTGCCATCTACCAC	AAATGGAACAGCTCCACAGC	82
PgPIP1-4	AGCTGCAGGACAGGTTTCAA	AGGACCGTCACGTAGAGGAA	105
PgPIP2-1	CATCGGAACCTTCGTCCTCG	ATCCCATGGCTTGTCCTTGT	95
PgPIP2-2	GAAGGCCTGGGATGACCAAT	CCGGAAGGACCCGAGCTT	94
PgPIP2-3	CCGCCGTGATCTACAACAAC	GCTGAAGGAGGCGGACGA	81
PgPIP2-5	CGCGACTCACACGTCCC	GACGATCTGGTGGTACAGCG	123
PgPIP2-6	CGACGGTGATCGGGTACAAG	CGCGAACGAGAGAGACCTT	115
PgPIP2-7	CGTCCTCGTGTACACCGTCT	ATCCAGTGGTTTTTCCATGC	97
PgPIP2-8	GGCGGCAAGAAGGACTACAA	AGCGTCACGTACACGAAGAG	92
*PgUBC**	TTCAAACCTCCGAAGGTGTCTT	GGCTCCACTGCTCTTTAAGAATG	89

PgUBC was used as a reference gene during the experiment. Primer efficiency was measured by RT-PCR reactions on five 1:10 cDNA serial dilutions. *From Reddy et al. (2017).
