# Supplementary material for: Aquaporins are main contributors to root hydraulic conductivity in pearl millet [Pennisetum glaucum (L) R. Br.]
Source: PLoS One. 2020 Oct 1;15(10):e0233481. doi: 10.1371/journal.pone.0233481 (PMC7529256; doi:10.1371/journal.pone.0233481)
Supplement: S5 Table — (PDF) [file pone.0233481.s005.pdf]

S5 Table. Root architectural traits in IP4952 and IP17150.

Line	Average root diameter (mm)	Total root length (cm)	Total root surface area (cm^2)
IP4952	0.20 ± 0.01	358.56 ± 27.23b	18.72 ± 1.21
IP17150	0.19 ± 0.01	477.08 ± 37.21a	20.26 ± 1.50
p-value	0.129	<0.05	0.417

Root diameter, root length and root surface area were measured using WinRhizo after root scanning on plants subjected to Lo measurements. Values represent mean ± se of n=25 plants.
