# Supplementary material for: Aquaporins are main contributors to root hydraulic conductivity in pearl millet [Pennisetum glaucum (L) R. Br.]
Source: PLoS One. 2020 Oct 1;15(10):e0233481. doi: 10.1371/journal.pone.0233481 (PMC7529256; doi:10.1371/journal.pone.0233481)
Supplement: S6 Table — (PDF) [file pone.0233481.s006.pdf]

**S6 Table. Root conductance ($L_0$), root hydraulic conductivity (Lpr) and aquaporin (AQP) contribution in IP4952 and IP17150.**

| Line | Azide | $L_0$ ($m^3\ s^{-1}\ MPa^{-1}$) | Lpr ($m^3\ m^{-2}\ s^{-1}\ MPa^{-1}$) | AQP contribution (%) |
|---|---|---|---|---|
| IP4952 | - | 3.78E-10 ± 5.24E-11a | 1.30E-07 ± 2.36E-08a | 84.64 ± 1.98a |
| | + | 6.27E-11 ± 1.24E-11c | 2.00E-08 ± 2.44E-09b | |
| IP17150 | - | 4.84E-10 ± 8.83E-11a | 9.27E-08 ± 8.33E-09a | 76.40 ± 2.61b |
| | + | 1.03E-10 ± 1.40E-11b | 2.19E-08 ± 2.32E-09b | |
| p-value | | <0.001 | <0.001 | <0.05 |

$L_0$ and Lpr were measured on plants grown in hydroponic conditions between 9AM to 12PM in absence (-) or presence (+) of 2mM azide. AQP contribution to Lpr in percent was calculated as relative Lpr inhibition by azide. Values represent mean ± se of n=10-15 plants.
