# Supplementary material for: Aquaporins are main contributors to root hydraulic conductivity in pearl millet [Pennisetum glaucum (L) R. Br.]
Source: PLoS One. 2020 Oct 1;15(10):e0233481. doi: 10.1371/journal.pone.0233481 (PMC7529256; doi:10.1371/journal.pone.0233481)
Supplement: S7 Table — (PDF) [file pone.0233481.s007.pdf]

S7 Table. Analysis of aquaporin conserved domains in pearl millet.

Query	PSSM-ID	E-Value	BitScore	Accession	Short name	SuperFamily
**Plasma membrane intrinsic proteins (PIP)**						
PgPIP1-1	333943	2.48e-97	284.98	pfam00230	MIP	-
PgPIP1-3	333943	5.26e-98	286.90	pfam00230	MIP	-
PgPIP1-4	333943	8.42e-87	258.78	pfam00230	MIP	-
PgPIP2-1	333943	4.11e-91	269.18	pfam00230	MIP	-
PgPIP2-2	333943	2.17e-89	264.95	pfam00230	MIP	-
PgPIP2-3	333943	3.23e-92	272.27	pfam00230	MIP	-
PgPIP2-5	333943	1.17e-83	249.92	pfam00230	MIP	-
PgPIP2-6	333943	1.50e-89	265.33	pfam00230	MIP	-
PgPIP2-7	333943	3.11e-84	251.85	pfam00230	MIP	-
PgPIP2-8	333943	5.37e-88	261.10	pfam00230	MIP	-
**Tonoplast intrinsic proteins (TIP)**						
PgTIP1-1	177664	1.73e-147	411.49	PLN00027	MIP	-
PgTIP2-1	350945	6.06e-122	346.92	cl00200	MIP	PLN00166
PgTIP2-2	350945	4.31e-104	301.86	cl00200	MIP	PLN00166
PgTIP2-3	350945	1.84e-119	340.76	cl00200	MIP	PLN00166
PgTIP3-1	350945	8.80e-89	263.57	cl00200	MIP	PLN00027
PgTIP4-1	350945	1.00e-65	204.25	cl00200	MIP	PLN00027
PgTIP4-2	350945	3.79e-62	195.00	cl00200	MIP	PLN00027
PgTIP4-3	350945	5.14e-68	210.03	cl00200	MIP	PLN00027
PgTIP5-1	350945	2.29e-64	201.67	cl00200	MIP	PLN00167
**Noduline-26 like intrinsic proteins (NIP)**						
PgNIP1-2	350945	8.22e-177	336.99	cl00200	MIP	PLN00184
PgNIP1-1	350945	1.32e-119	344.31	cl00200	MIP	PLN00184
PgNIP1-4	350945	7.80e-88	262.92	cl00200	MIP	PLN00182
PgNIP2-2	350945	9.10e-75	230.90	cl00200	MIP	PLN00026
PgNIP2-1	350945	1.78e-78	240.15	cl00200	MIP	PLN00026
PgNIP3-1	177663	5.01e-174	482.82	PLN00026	MIP	-
PgNIP3-2	350945	1.52e-85	257.48	cl00200	MIP	PLN00026
PgNIP3-3	350945	5.02e-85	255.56	cl00200	MIP	PLN00026
PgNIP3-4	350945	1.14e-59	190.46	cl00200	MIP	PLN00026
PgNIP4-1	350945	1.09e-37	135.04	cl00200	MIP	PLN00182
PgNIP3-5	350945	6.46e-49	163.88	cl00200	MIP	PLN00026
**Small intrinsic proteins (SIP)**						
PgSIP1-1	350945	4.91e-14	68.82	cl00200	MIP	cd00333
PgSIP1-2	350945	4.51e-11	60.73	cl00200	MIP	cd00333
PgSIP2-1	350945	5.02e-09	54.61	cl00200	MIP	TIGR00861

The unique identifier for the position-specific scoring matrix (PSSM-ID) generated by the sequence alignment with its p-value, the bit-score, the accession number of the hit, the short name of the conserved domain and the superfamily to which it belongs for each isoforms were obtained using the NCBI conserved domain database (CDD).
