# Supplementary material for: Aquaporins are main contributors to root hydraulic conductivity in pearl millet [Pennisetum glaucum (L) R. Br.]
Source: PLoS One. 2020 Oct 1;15(10):e0233481. doi: 10.1371/journal.pone.0233481 (PMC7529256; doi:10.1371/journal.pone.0233481)
Supplement: S8 Table — (PDF) [file pone.0233481.s008.pdf]

**S8 Table. Transmembrane domain analysis of aquaporins in pearl millet.**

| Gene | TMHMM | | | | | TMPred | | | Phyre2 | | |
|---|---|---|---|---|---|---|---|---|---|---|---|
| | Exp. AA | First 60 | N-ter | C-ter | TM | N-ter | C-ter | TM | N-ter | C-ter | TM |
| **Plasma membrane intrinsic proteins (PIP)** | | | | | | | | | | | |
| PgPIP1-1 | 130.8 | 5.35 | in | in | 6 | in | in | 6 | in | in | 6 |
| PgPIP1-3 | 129.6 | 5.40 | in | in | 6 | In | in | 6 | in | in | 6 |
| PgPIP1-4 | 132.9 | 0.25 | in | in | 6 | in | in | 6 | in | in | 6 |
| PgPIP2-1 | 141.4 | 17.92 | out | out | 6 | in | in | 6 | in | in | 6 |
| PgPIP2-2 | 135.3 | 18.48 | in | in | 6 | in | in | 6 | in | in | 6 |
| PgPIP2-3 | 133.3 | 17.67 | in | in | 6 | in | in | 6 | in | in | 6 |
| PgPIP2-5 | 136.9 | 20.82 | in | in | 6 | in | in | 6 | in | in | 6 |
| PgPIP2-6 | 139.3 | 20.81 | out | in | 7 | in | in | 6 | in | in | 6 |
| PgPIP2-7 | 140.9 | 17.23 | in | in | 6 | in | in | 6 | in | in | 6 |
| PgPIP2-8 | 132.8 | 21.99 | in | in | 6 | in | in | 6 | in | in | 6 |
| **Tonoplast intrinsic proteins (TIP)** | | | | | | | | | | | |
| PgTIP1-1 | 137.1 | 26.29 | in | in | 6 | in | in | 6 | in | in | 6 |
| PgTIP2-3 | 136.7 | 28.76 | out | out | 6 | in | in | 6 | in | in | 6 |
| PgTIP2-1 | 137.0 | 29.24 | out | out | 6 | in | in | 6 | in | in | 6 |
| PgTIP4-1 | 133.0 | 25.54 | in | in | 6 | in | in | 6 | in | in | 6 |
| PgTIP3-1 | 134.4 | 20.98 | in | in | 6 | in | out | 6 | in | in | 6 |
| PgTIP2-2 | 142.1 | 26.95 | in | in | 6 | in | in | 6 | in | in | 6 |
| PgTIP4-3 | 132.9 | 29.79 | in | in | 6 | in | out | 6 | in | in | 6 |
| PgTIP4-2 | 138.1 | 33.59 | in | in | 6 | in | out | 6 | in | in | 6 |
| PgTIP5-1 | 132.56 | 21.99 | in | in | 6 | out | out | 6 | in | in | 6 |
| **Noduline-26 like intrinsic proteins (NIP)** | | | | | | | | | | | |
| PgNIP1-2 | 122.1 | 9.84 | out | in | 5 | in | in | 6 | in | in | 6 |
| PgNIP1-1 | 128.3 | 16.10 | in | in | 6 | in | in | 6 | in | in | 6 |
| PgNIP1-4 | 131.4 | 0.40 | in | in | 6 | in | in | 6 | in | in | 6 |
| PgNIP2-2 | 129.4 | 4.75 | in | in | 6 | in | in | 6 | in | in | 6 |
| PgNIP2-1 | 132.9 | 9.80 | in | in | 6 | in | in | 6 | in | in | 6 |
| PgNIP3-1 | 122.3 | 0.11 | out | in | 5 | in | in | 6 | in | in | 6 |
| PgNIP3-2 | 127.3 | 9.76 | in | in | 6 | in | in | 6 | in | in | 6 |
| PgNIP3-3 | 129.6 | 9.98 | in | in | 6 | in | in | 6 | in | in | 6 |
| PgNIP3-4 | 129.82 | 20.04 | in | in | 6 | in | in | 6 | in | in | 6 |
| PgNIP4-1 | 130.3 | 3.05 | in | in | 6 | in | out | 7 | in | in | 6 |
| PgNIP3-5 | 125.11 | 0.006 | in | in | 6 | in | in | 6 | in | in | 6 |
| **Small intrinsic proteins (SIPs)** | | | | | | | | | | | |
| PgSIP1-1 | 133.1 | 41.61 | in | in | 6 | in | out | 7 | in | in | 6 |
| PglSIP1-2 | 130.1 | 29.71 | in | in | 6 | out | in | 7 | out | out | 6 |
| PgSIP2-1 | 131.4 | 26.07 | out | in | 5 | in | out | 7 | in | in | 6 |

Cytosolic (in) or extra-cellular (out) location of the N-terminal (N-ter) and C-terminal (C-ter) ends of the different aquaporin isoforms as well as the number of transmembrane domains (TM) were predicted using TMHMM, TMPred and Phyre2 software. TMHMM provided information on the expected total number of amino-acids in the transmembrane domains (Exp. AA) and the number of amino-acids of the N-ter region (First 60).
