# Supplementary material for: Aquaporins are main contributors to root hydraulic conductivity in pearl millet [Pennisetum glaucum (L) R. Br.]
Source: PLoS One. 2020 Oct 1;15(10):e0233481. doi: 10.1371/journal.pone.0233481 (PMC7529256; doi:10.1371/journal.pone.0233481)

S1 Figure. Reversion of azide-induced root hydraulic conductivity inhibition. Root hydraulic conductivity (Lpr) were measured between 9AM to 1PM in Sauna3 pearl millet plants grown in hydroponic conditions. Measurements were performed in the nutrient solution without azide (no azide), in the presence of 2mM azide (with azide) and after exposing roots from plants subjected to Lpr measurements in the presence of azide to the nutrient solution without azide (recovery). Bars represent mean values ± se of n=4 plants.

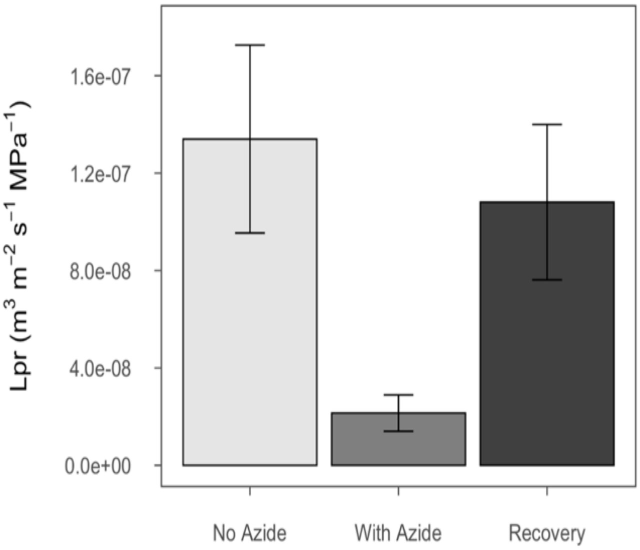

Supplement: S1 Fig — (PDF) [file pone.0233481.s009.pdf]
