# Supplementary material for: Aquaporins are main contributors to root hydraulic conductivity in pearl millet [Pennisetum glaucum (L) R. Br.]
Source: PLoS One. 2020 Oct 1;15(10):e0233481. doi: 10.1371/journal.pone.0233481 (PMC7529256; doi:10.1371/journal.pone.0233481)

**S2 Figure. Structure of pearl millet aquaporins genes.** Exon and intron representation of the 33 aquaporins identified in pearl millet was obtained using GSDS2.0. Full length open reading frames are represented for each genes in base pair (bp) from 5' to 3', with exon in bleu boxes and intron in black lines.

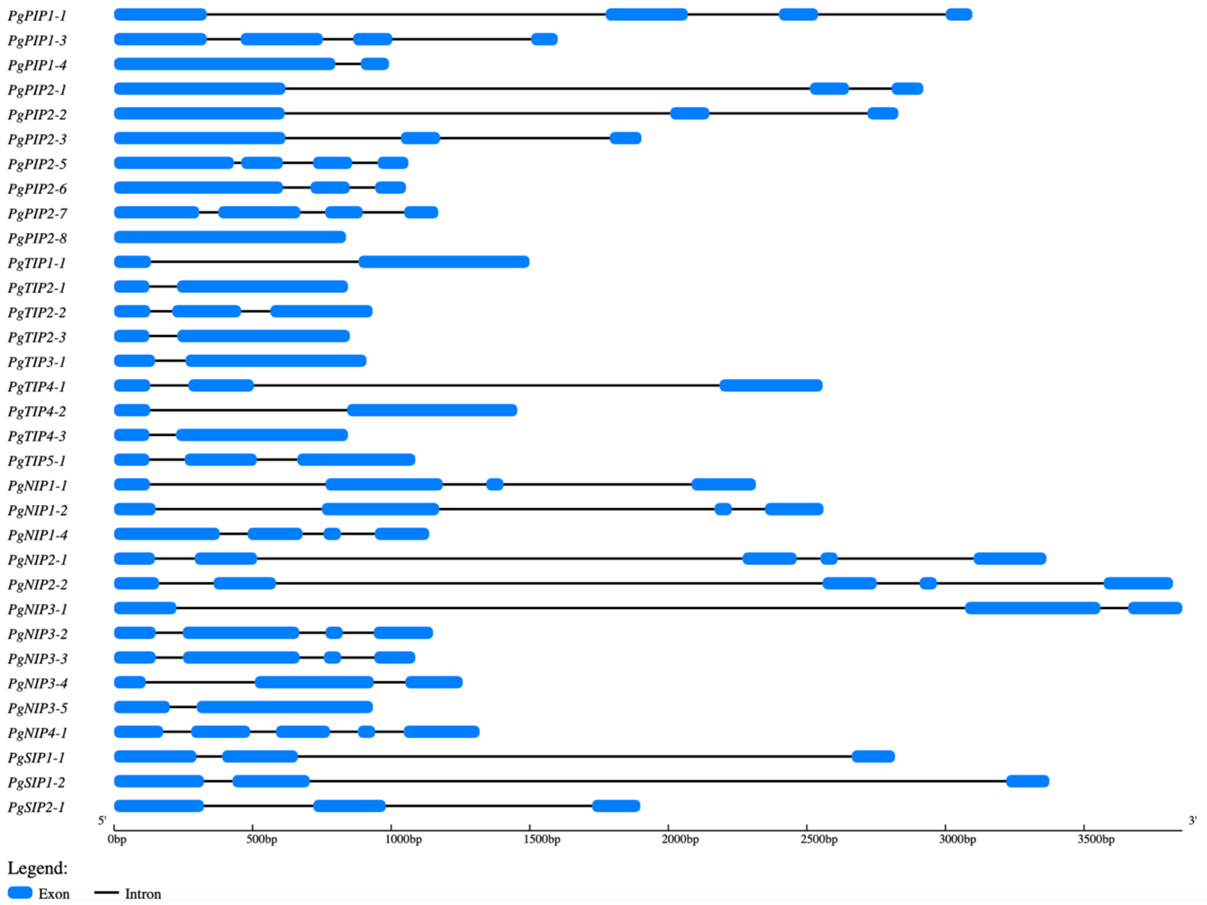

Supplement: S2 Fig — (PDF) [file pone.0233481.s010.pdf]
