# Supplementary material for: Aquaporins are main contributors to root hydraulic conductivity in pearl millet [Pennisetum glaucum (L) R. Br.]
Source: PLoS One. 2020 Oct 1;15(10):e0233481. doi: 10.1371/journal.pone.0233481 (PMC7529256; doi:10.1371/journal.pone.0233481)

S3 Figure. Conserved domains and membrane topology of the TIP isoforms from pearl millet. Alignment of the TIP isoforms were obtained using ClustalW in Mega7. Sequence identities and similarities (80%) are highlighted in colors. The transmembrane domains are represented by orange bars and the N-terminal and C-terminal ends of the protein are located in the cytosol. NPA: Asparagine-Proline-Alanine motifs; *: Aromatic/Arginine selectivity filters; #: Froger's residues.

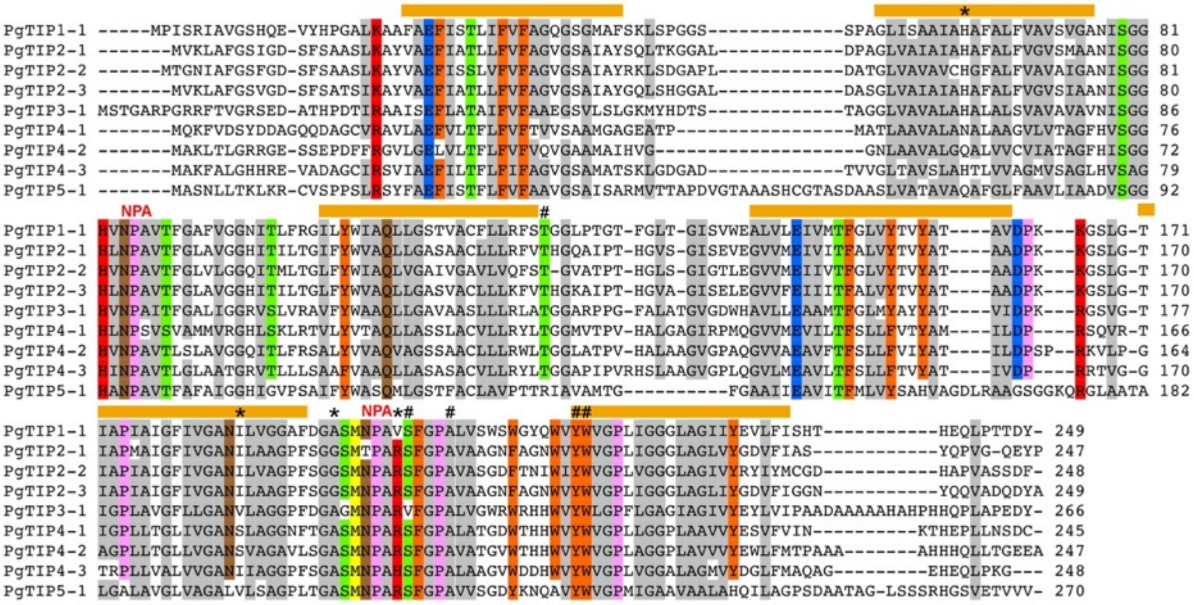

Supplement: S3 Fig — (PDF) [file pone.0233481.s011.pdf]
