# Supplementary material for: Aquaporins are main contributors to root hydraulic conductivity in pearl millet [Pennisetum glaucum (L) R. Br.]
Source: PLoS One. 2020 Oct 1;15(10):e0233481. doi: 10.1371/journal.pone.0233481 (PMC7529256; doi:10.1371/journal.pone.0233481)

S6 Figure. Cavity features of PgPIP isoforms. Three-dimensional geometry structure and pore morphology was obtained using the PoreWalker software from the EMBL-EBI laboratory. Protein is oriented with lowest coordinate along the pore axis (=x axis) at the bottom. XY-plane section with Z<0 and coordinates only are represented. Red shapes represent pore centers at 1 Angstrom step along the pore axis.

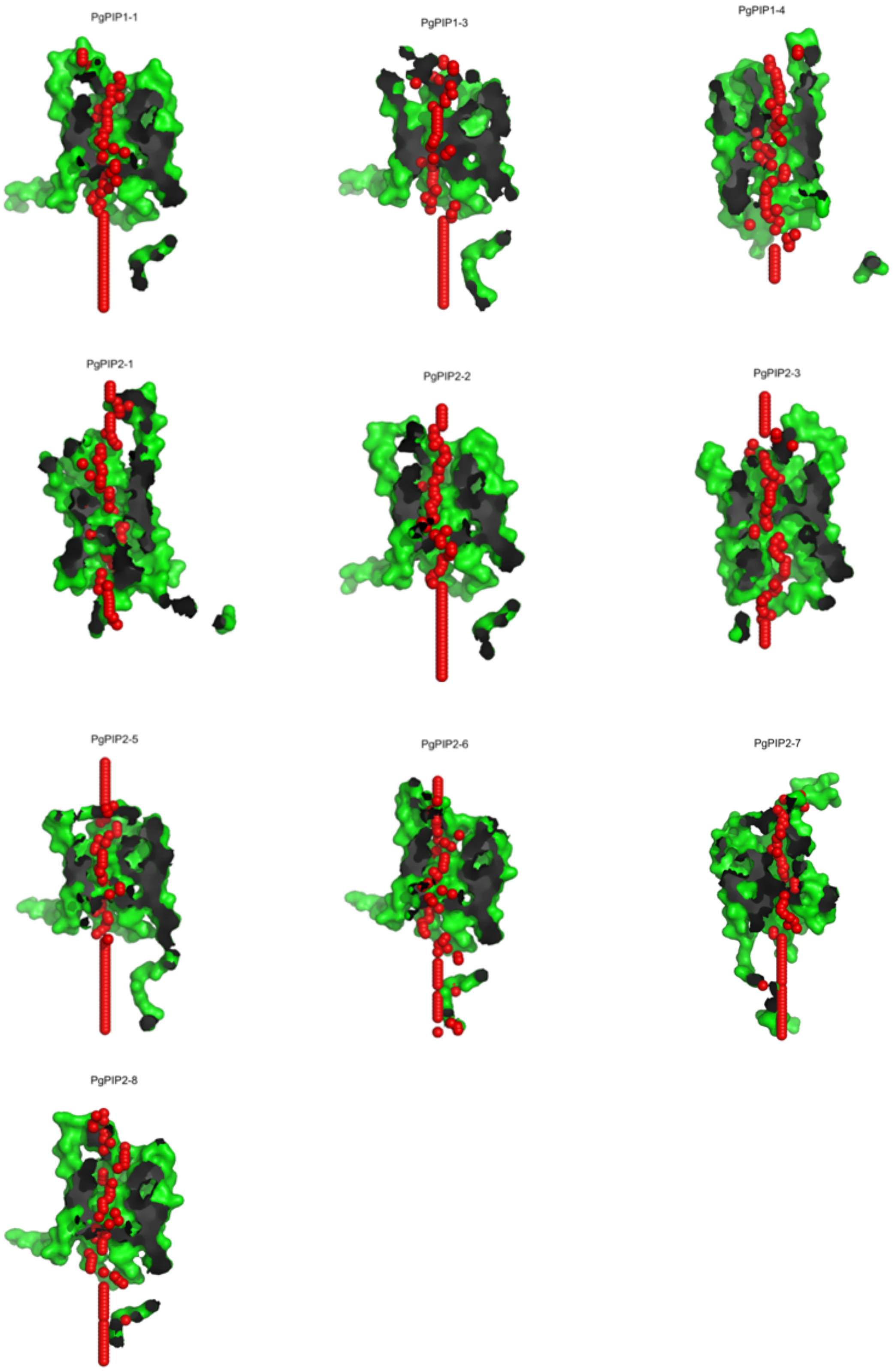

Supplement: S6 Fig — (PDF) [file pone.0233481.s014.pdf]
