# Supplementary material for: Aquaporins are main contributors to root hydraulic conductivity in pearl millet [Pennisetum glaucum (L) R. Br.]
Source: PLoS One. 2020 Oct 1;15(10):e0233481. doi: 10.1371/journal.pone.0233481 (PMC7529256; doi:10.1371/journal.pone.0233481)

S7 Figure. Expression pattern of PgPIP isoforms in shoots of pearl millet. Shoot transcriptomic data (leaves and inflorescence) were retrieved from Sarah et al., (2017). Bars represent the mean ± se of transcript abundance in reads from n=10 transcriptomes, each from different pearl millet varieties.

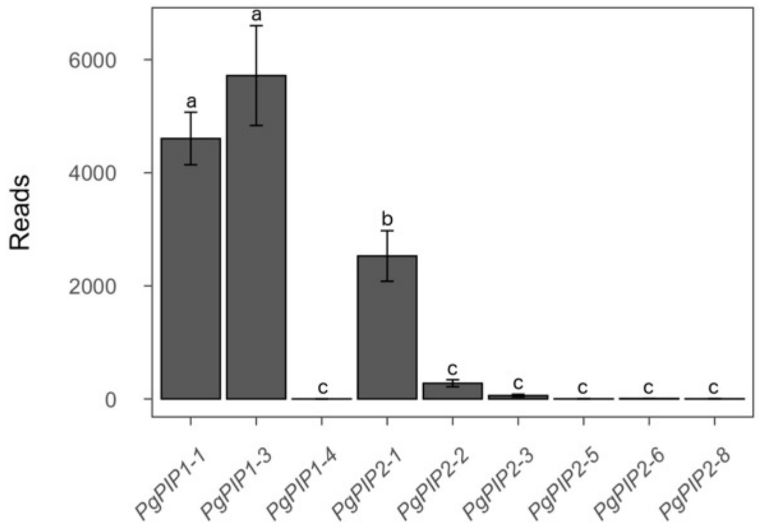

Supplement: S7 Fig — (PDF) [file pone.0233481.s015.pdf]
